# Supplementary material for: Genome-wide CRISPR off-target prediction and optimization using RNA-DNA interaction fingerprints
Source: Nat Commun. 2023 Nov 18;14:7521. doi: 10.1038/s41467-023-42695-4 (PMC10657421; doi:10.1038/s41467-023-42695-4)
Supplement: Supplementary file 1 — Supplementary Information [file 41467_2023_42695_MOESM1_ESM.pdf]

# Supplementary Information for
# "Genome-wide CRISPR off-target prediction and optimization using RNA-DNA interaction fingerprints"

Qinchang Chen[1, 3, #], Guohui Chuai[2, #], Haihang Zhang[6, #], Jin Tang[3], Liwen Duan[3], Huan Guan[3], Wenhui Li[3], Wannian Li[1], Jiaying Wen[1], Erwei Zuo[6, *], Qing Zhang[5, 7, *], Qi Liu[1,2,3,4, *]

1 Key Laboratory of Spine and Spinal Cord Injury Repair and Regeneration (Tongji University), Ministry of Education, Orthopaedic Department of Tongji Hospital, Frontier Science Center for Stem Cell Research, Bioinformatics Department, School of Life Sciences and Technology, Tongji University, Shanghai, 200092, China
2 Translational Medical Center for Stem Cell Therapy and Institute for Regenerative Medicine, Shanghai East Hospital, Frontier Science Center for Stem Cell Research, Bioinformatics Department, School of Life Sciences and Technology, Tongji University, Shanghai, 200092, China.
3 Research Institute of Intelligent Computing, Zhejiang Lab, Hangzhou 311121, China.
4 Shanghai Research Institute for Intelligent Autonomous Systems, Shanghai 201210, China.
5 Roche R&D Center (China) Ltd., China Innovation Center of Roche, Shanghai, 201203, China
6 Shenzhen Branch, Guangdong Laboratory of Lingnan Modern Agriculture, Key Laboratory of Gene Editing Technologies(Hainan), Ministry of Agriculture and Rural Affairs, Agricultural Genomics Institute at Shenzhen, Chinese Academy of Agricultural Sciences, Shenzhen, China
7 Current address: Ailomics Therapeutics, Shanghai, 201203, China

# These authors contributed equally.
* Corresponding authors

*Correspondence: qiliu@tongji.edu.cn (Q. Liu), qing.zhang@ailomics.com (Q. Zhang) and zuoerwei@caas.cn (E. Zuo)

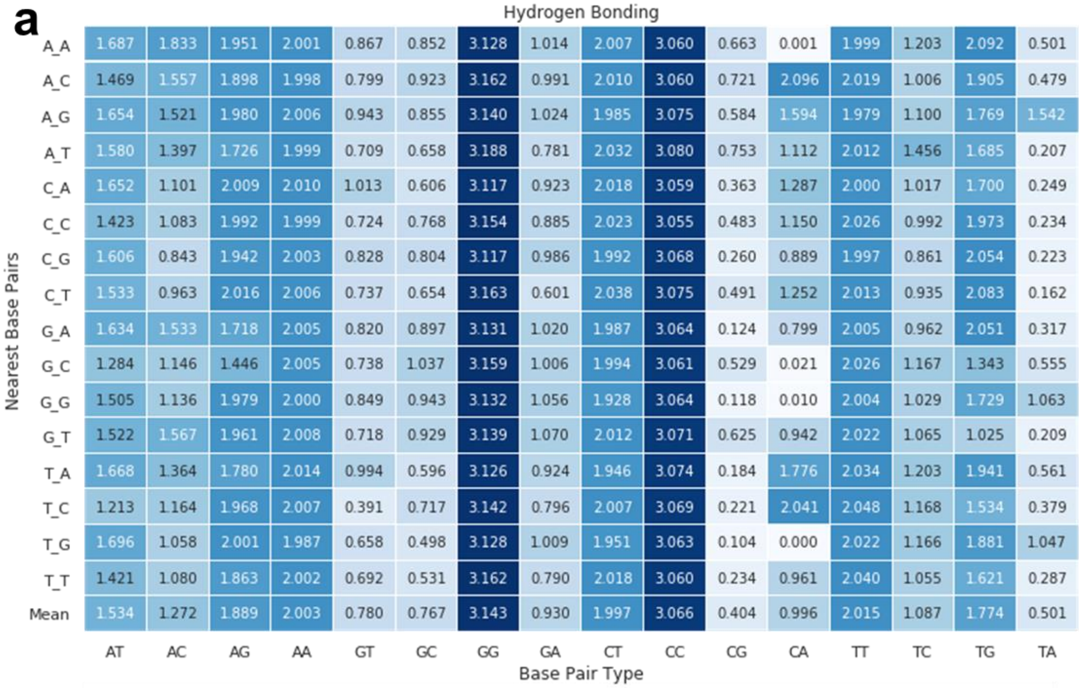
a
Hydrogen Bonding
Nearest Base Pairs
Base Pair Type
AT AC AG AA GT GC GG GA CT CC CG CA TT TC TG TA
A_A 1.687 1.833 1.951 2.001 0.867 0.852 3.128 1.014 2.007 3.060 0.663 0.001 1.999 1.203 2.092 0.501
A_C 1.469 1.557 1.898 1.998 0.799 0.923 3.162 0.991 2.010 3.060 0.721 2.096 2.019 1.006 1.905 0.479
A_G 1.654 1.521 1.980 2.006 0.943 0.855 3.140 1.024 1.985 3.075 0.584 1.594 1.979 1.100 1.769 1.542
A_T 1.580 1.397 1.726 1.999 0.709 0.658 3.188 0.781 2.032 3.080 0.753 1.112 2.012 1.456 1.685 0.207
C_A 1.652 1.101 2.009 2.010 1.013 0.606 3.117 0.923 2.018 3.059 0.363 1.287 2.000 1.017 1.700 0.249
C_C 1.423 1.083 1.992 1.999 0.724 0.768 3.154 0.885 2.023 3.055 0.483 1.150 2.026 0.992 1.973 0.234
C_G 1.606 0.843 1.942 2.003 0.828 0.804 3.117 0.986 1.992 3.068 0.260 0.889 1.997 0.861 2.054 0.223
C_T 1.533 0.963 2.016 2.006 0.737 0.654 3.163 0.601 2.038 3.075 0.491 1.252 2.013 0.935 2.083 0.162
G_A 1.634 1.533 1.718 2.005 0.820 0.897 3.131 1.020 1.987 3.064 0.124 0.799 2.005 0.962 2.051 0.317
G_C 1.284 1.146 1.446 2.005 0.738 1.037 3.159 1.006 1.994 3.061 0.529 0.021 2.026 1.167 1.343 0.555
G_G 1.505 1.136 1.979 2.000 0.849 0.943 3.132 1.056 1.928 3.064 0.118 0.010 2.004 1.029 1.729 1.063
G_T 1.522 1.567 1.961 2.008 0.718 0.929 3.139 1.070 2.012 3.071 0.625 0.942 2.022 1.065 1.025 0.209
T_A 1.668 1.364 1.780 2.014 0.994 0.596 3.126 0.924 1.946 3.074 0.184 1.776 2.034 1.203 1.941 0.561
T_C 1.213 1.164 1.968 2.007 0.391 0.717 3.142 0.796 2.007 3.069 0.221 2.041 2.048 1.168 1.534 0.379
T_G 1.696 1.058 2.001 1.987 0.658 0.498 3.128 1.009 1.951 3.063 0.104 0.000 2.022 1.166 1.881 1.047
T_T 1.421 1.080 1.863 2.002 0.692 0.531 3.162 0.790 2.018 3.060 0.234 0.961 2.040 1.055 1.621 0.287
Mean 1.534 1.272 1.889 2.003 0.780 0.767 3.143 0.930 1.997 3.066 0.404 0.996 2.015 1.087 1.774 0.501

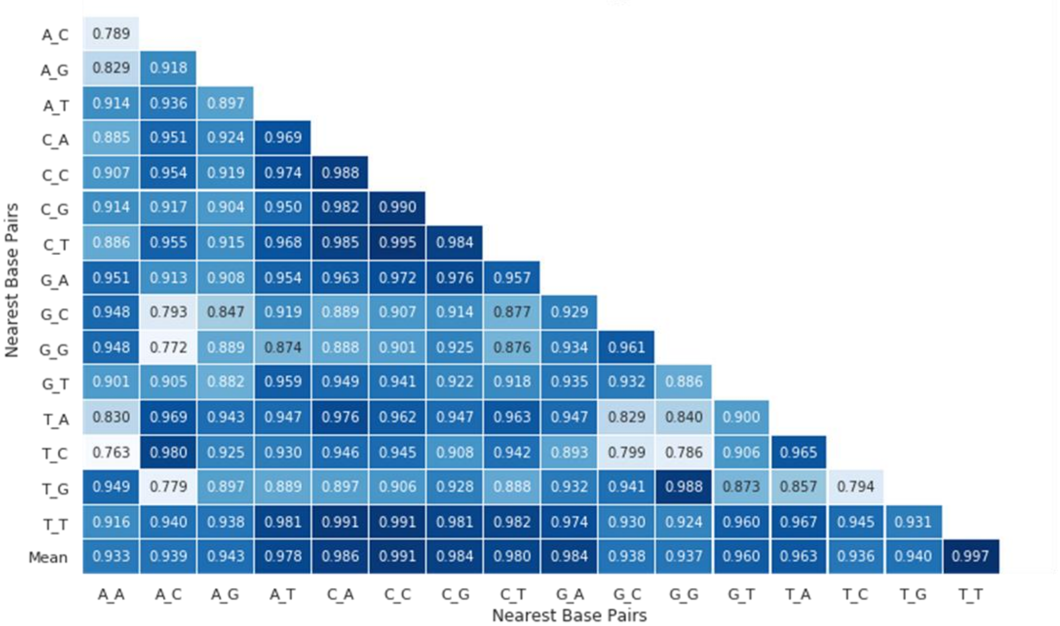
Nearest Base Pairs
Nearest Base Pairs
A_A A_C A_G A_T C_A C_C C_G C_T G_A G_C G_G G_T T_A T_C T_G T_T
A_C 0.789
A_G 0.829 0.918
A_T 0.914 0.936 0.897
C_A 0.885 0.951 0.924 0.969
C_C 0.907 0.954 0.919 0.974 0.988
C_G 0.914 0.917 0.904 0.950 0.982 0.990
C_T 0.886 0.955 0.915 0.968 0.985 0.995 0.984
G_A 0.951 0.913 0.908 0.954 0.963 0.972 0.976 0.957
G_C 0.948 0.793 0.847 0.919 0.889 0.907 0.914 0.877 0.929
G_G 0.948 0.772 0.889 0.874 0.888 0.901 0.925 0.876 0.934 0.961
G_T 0.901 0.905 0.882 0.959 0.949 0.941 0.922 0.918 0.935 0.932 0.886
T_A 0.830 0.969 0.943 0.947 0.976 0.962 0.947 0.963 0.947 0.829 0.840 0.900
T_C 0.763 0.980 0.925 0.930 0.946 0.945 0.908 0.942 0.893 0.799 0.786 0.906 0.965
T_G 0.949 0.779 0.897 0.889 0.897 0.906 0.928 0.888 0.932 0.941 0.988 0.873 0.857 0.794
T_T 0.916 0.940 0.938 0.981 0.991 0.991 0.981 0.982 0.974 0.930 0.924 0.960 0.967 0.945 0.931
Mean 0.933 0.939 0.943 0.978 0.986 0.991 0.984 0.980 0.984 0.938 0.937 0.960 0.963 0.936 0.940 0.997

**b**

Free Energy Contribution of RNA Nucleotide

| Nearest Base Pairs | AT | AC | AG | AA | GT | GC | GG | GA | CT | CC | CG | CA | TT | TC | TG | TA |
|---|---|---|---|---|---|---|---|---|---|---|---|---|---|---|---|---|
| A_A | -2.159 | -2.039 | -3.578 | -2.036 | 0.067 | 0.145 | -3.920 | 0.276 | -4.171 | -7.252 | -0.940 | -0.575 | -3.578 | -1.630 | -3.620 | -0.570 |
| A_C | -1.708 | -1.442 | -3.284 | -2.194 | 0.251 | -0.075 | -3.858 | 0.491 | -4.378 | -7.362 | -1.115 | -1.552 | -3.748 | -1.669 | -2.987 | -0.299 |
| A_G | -2.032 | -1.321 | -3.508 | -1.973 | -0.088 | -0.008 | -3.882 | 0.358 | -3.712 | -6.707 | -0.330 | -0.819 | -3.909 | -1.425 | -2.732 | -1.428 |
| A_T | -1.853 | -1.200 | -2.578 | -2.254 | 0.372 | -0.214 | -3.695 | 0.461 | -4.336 | -7.513 | -0.885 | -1.065 | -3.674 | -1.436 | -2.174 | -0.395 |
| C_A | -2.138 | -0.934 | -3.353 | -1.805 | -0.007 | 0.291 | -3.999 | 0.239 | -4.119 | -7.122 | -0.295 | -1.147 | -3.225 | -1.251 | -2.685 | -0.294 |
| C_C | -1.826 | -0.711 | -3.173 | -1.897 | 0.291 | -0.296 | -3.828 | 0.547 | -4.299 | -7.388 | -0.367 | -1.002 | -3.191 | -1.834 | -2.974 | -0.373 |
| C_G | -1.826 | -0.286 | -2.990 | -1.671 | 0.008 | -0.007 | -4.034 | 0.281 | -3.696 | -6.778 | 0.482 | -0.291 | -3.475 | -1.915 | -3.294 | -0.207 |
| C_T | -2.011 | -0.639 | -3.212 | -1.967 | 0.280 | 0.025 | -3.748 | 0.578 | -4.335 | -7.459 | -0.418 | -1.214 | -3.166 | -1.259 | -3.098 | -0.175 |
| G_A | -2.438 | -1.772 | -3.044 | -1.994 | -0.640 | -0.515 | -4.780 | -0.170 | -4.137 | -7.220 | -1.976 | -1.242 | -3.921 | -1.563 | -3.769 | -0.979 |
| G_C | -1.776 | -0.936 | -2.070 | -2.005 | -0.320 | -0.431 | -4.628 | 0.333 | -4.385 | -7.179 | -1.008 | -0.785 | -3.998 | -1.390 | -2.288 | -1.065 |
| G_G | -2.162 | -1.322 | -3.832 | -1.855 | -0.708 | -0.386 | -4.837 | -0.129 | -3.681 | -6.588 | -0.732 | -0.256 | -4.180 | -1.529 | -3.291 | -1.289 |
| G_T | -2.156 | -1.357 | -3.657 | -2.182 | -0.266 | -0.490 | -4.433 | -0.085 | -4.309 | -7.374 | -1.009 | -1.079 | -3.948 | -1.477 | -1.552 | -0.868 |
| T_A | -1.829 | -0.867 | -2.427 | -1.448 | 0.215 | 1.066 | -3.584 | 0.565 | -3.074 | -6.229 | 0.752 | -1.741 | -3.096 | -0.170 | -3.014 | -0.138 |
| T_C | -1.420 | -0.529 | -2.919 | -1.538 | 0.684 | 0.384 | -3.580 | 0.555 | -3.483 | -6.455 | 0.819 | -1.442 | -3.159 | -0.423 | -2.190 | 0.013 |
| T_G | -1.816 | -0.040 | -2.956 | -1.284 | 0.400 | 0.413 | -3.674 | 0.613 | -2.836 | -5.871 | 1.484 | 1.053 | -3.311 | -0.677 | -2.964 | -0.812 |
| T_T | -1.594 | -0.370 | -2.501 | -1.625 | 0.547 | 0.568 | -3.433 | 0.645 | -3.454 | -6.551 | 0.769 | -0.407 | -3.120 | -0.343 | -2.106 | 0.064 |
| Mean | -1.922 | -0.985 | -3.068 | -1.858 | 0.068 | 0.030 | -3.994 | 0.347 | -3.900 | -6.941 | -0.298 | -0.848 | -3.544 | -1.249 | -2.796 | -0.551 |

Base Pair Type

| Nearest Base Pairs | A_A | A_C | A_G | A_T | C_A | C_C | C_G | C_T | G_A | G_C | G_G | G_T | T_A | T_C | T_G | T_T |
|---|---|---|---|---|---|---|---|---|---|---|---|---|---|---|---|---|
| A_C | 0.981 | | | | | | | | | | | | | | | |
| A_G | 0.977 | 0.971 | | | | | | | | | | | | | | |
| A_T | 0.968 | 0.990 | 0.968 | | | | | | | | | | | | | |
| C_A | 0.980 | 0.986 | 0.981 | 0.980 | | | | | | | | | | | | |
| C_C | 0.977 | 0.988 | 0.975 | 0.984 | 0.990 | | | | | | | | | | | |
| C_G | 0.965 | 0.958 | 0.971 | 0.951 | 0.976 | 0.982 | | | | | | | | | | |
| C_T | 0.978 | 0.990 | 0.973 | 0.983 | 0.995 | 0.996 | 0.975 | | | | | | | | | |
| G_A | 0.979 | 0.974 | 0.961 | 0.967 | 0.972 | 0.968 | 0.954 | 0.972 | | | | | | | | |
| G_C | 0.944 | 0.962 | 0.961 | 0.980 | 0.961 | 0.964 | 0.949 | 0.960 | 0.970 | | | | | | | |
| G_G | 0.966 | 0.943 | 0.981 | 0.936 | 0.963 | 0.954 | 0.972 | 0.950 | 0.965 | 0.951 | | | | | | |
| G_T | 0.949 | 0.967 | 0.970 | 0.980 | 0.975 | 0.965 | 0.940 | 0.963 | 0.948 | 0.970 | 0.952 | | | | | |
| T_A | 0.945 | 0.950 | 0.954 | 0.933 | 0.969 | 0.945 | 0.943 | 0.962 | 0.943 | 0.919 | 0.928 | 0.916 | | | | |
| T_C | 0.954 | 0.972 | 0.973 | 0.965 | 0.986 | 0.974 | 0.962 | 0.981 | 0.944 | 0.944 | 0.944 | 0.962 | 0.981 | | | |
| T_G | 0.936 | 0.898 | 0.963 | 0.897 | 0.936 | 0.931 | 0.970 | 0.926 | 0.916 | 0.910 | 0.976 | 0.906 | 0.916 | 0.928 | | |
| T_T | 0.970 | 0.974 | 0.984 | 0.977 | 0.992 | 0.981 | 0.977 | 0.986 | 0.962 | 0.966 | 0.966 | 0.973 | 0.972 | 0.990 | 0.955 | |
| Mean | 0.985 | 0.987 | 0.991 | 0.984 | 0.996 | 0.991 | 0.983 | 0.992 | 0.980 | 0.974 | 0.976 | 0.976 | 0.966 | 0.984 | 0.953 | 0.995 |

Nearest Base Pairs

**c**

Atom Distance between RNA@P and DNA@P

| Nearest Base Pairs \ Base Pair Type | AT | AC | AG | AA | GT | GC | GG | GA | CT | CC | CG | CA | TT | TC | TG | TA |
|---|---|---|---|---|---|---|---|---|---|---|---|---|---|---|---|---|
| A_A | 18.710 | 18.595 | 20.275 | 19.729 | 18.716 | 18.750 | 19.693 | 19.225 | 19.221 | 19.373 | 19.154 | 19.694 | 19.492 | 18.987 | 19.438 | 19.315 |
| A_C | 18.539 | 18.551 | 20.011 | 19.560 | 18.536 | 18.434 | 19.483 | 18.932 | 19.276 | 19.538 | 19.254 | 20.063 | 19.620 | 18.885 | 19.661 | 18.372 |
| A_G | 19.039 | 18.920 | 20.142 | 19.763 | 18.709 | 18.673 | 19.689 | 19.189 | 19.259 | 19.209 | 19.379 | 19.854 | 19.473 | 18.687 | 19.071 | 19.802 |
| A_T | 18.362 | 18.732 | 19.809 | 19.816 | 18.459 | 18.051 | 19.779 | 19.128 | 19.198 | 19.490 | 18.985 | 19.846 | 19.605 | 18.471 | 19.818 | 19.304 |
| C_A | 18.830 | 19.057 | 20.155 | 19.690 | 18.893 | 18.549 | 19.754 | 19.363 | 19.240 | 19.353 | 19.592 | 19.744 | 19.541 | 19.264 | 19.686 | 19.861 |
| C_C | 18.504 | 18.925 | 19.982 | 19.574 | 18.681 | 18.691 | 19.524 | 19.183 | 19.275 | 19.496 | 19.431 | 19.991 | 19.619 | 19.247 | 19.896 | 19.681 |
| C_G | 19.013 | 19.396 | 20.041 | 19.611 | 18.909 | 19.000 | 19.593 | 19.193 | 19.290 | 19.273 | 19.789 | 19.848 | 19.518 | 19.362 | 19.355 | 20.313 |
| C_T | 18.603 | 19.087 | 20.095 | 19.786 | 18.653 | 18.656 | 19.808 | 19.286 | 19.204 | 19.398 | 19.449 | 19.871 | 19.614 | 19.448 | 19.932 | 19.486 |
| G_A | 18.883 | 18.835 | 19.996 | 19.787 | 18.785 | 18.811 | 19.948 | 19.436 | 19.201 | 19.419 | 19.208 | 19.821 | 19.438 | 19.030 | 19.496 | 19.431 |
| G_C | 18.398 | 18.536 | 18.957 | 19.634 | 18.554 | 18.396 | 19.607 | 18.985 | 19.206 | 19.534 | 19.205 | 19.692 | 19.543 | 18.980 | 19.248 | 19.272 |
| G_G | 19.142 | 18.379 | 20.191 | 19.761 | 18.793 | 18.864 | 19.869 | 19.298 | 19.237 | 19.287 | 19.749 | 20.022 | 19.409 | 18.915 | 19.648 | 19.708 |
| G_T | 18.580 | 18.398 | 20.233 | 19.826 | 18.522 | 18.574 | 19.865 | 19.110 | 19.130 | 19.526 | 18.885 | 19.972 | 19.524 | 19.138 | 19.310 | 19.250 |
| T_A | 18.701 | 18.853 | 19.967 | 19.609 | 18.537 | 17.823 | 19.577 | 19.040 | 19.317 | 19.380 | 19.887 | 19.476 | 19.494 | 19.255 | 19.372 | 19.993 |
| T_C | 18.385 | 19.088 | 20.058 | 19.441 | 18.422 | 18.400 | 19.454 | 18.697 | 19.251 | 19.492 | 19.828 | 19.978 | 19.573 | 19.576 | 19.587 | 19.497 |
| T_G | 18.912 | 19.452 | 20.177 | 19.562 | 18.565 | 18.426 | 19.682 | 19.104 | 19.328 | 19.304 | 20.137 | 20.309 | 19.444 | 19.393 | 19.612 | 19.925 |
| T_T | 18.462 | 18.741 | 19.852 | 19.672 | 18.367 | 18.240 | 19.747 | 18.901 | 19.250 | 19.370 | 19.939 | 20.007 | 19.528 | 19.384 | 20.043 | 19.852 |
| Mean | 18.691 | 18.846 | 19.996 | 19.676 | 18.631 | 18.521 | 19.692 | 19.129 | 19.243 | 19.403 | 19.492 | 19.887 | 19.527 | 19.126 | 19.573 | 19.566 |

| Nearest Base Pairs | A_A | A_C | A_G | A_T | C_A | C_C | C_G | C_T | G_A | G_C | G_G | G_T | T_A | T_C | T_G | T_T |
|---|---|---|---|---|---|---|---|---|---|---|---|---|---|---|---|---|
| A_C | 0.849 | | | | | | | | | | | | | | | |
| A_G | 0.872 | 0.641 | | | | | | | | | | | | | | |
| A_T | 0.886 | 0.843 | 0.809 | | | | | | | | | | | | | |
| C_A | 0.888 | 0.703 | 0.870 | 0.876 | | | | | | | | | | | | |
| C_C | 0.875 | 0.809 | 0.768 | 0.901 | 0.926 | | | | | | | | | | | |
| C_G | 0.657 | 0.386 | 0.831 | 0.607 | 0.852 | 0.766 | | | | | | | | | | |
| C_T | 0.892 | 0.827 | 0.750 | 0.900 | 0.933 | 0.959 | 0.716 | | | | | | | | | |
| G_A | 0.962 | 0.810 | 0.880 | 0.920 | 0.888 | 0.857 | 0.653 | 0.895 | | | | | | | | |
| G_C | 0.721 | 0.736 | 0.656 | 0.854 | 0.730 | 0.807 | 0.541 | 0.780 | 0.782 | | | | | | | |
| G_G | 0.893 | 0.732 | 0.880 | 0.793 | 0.861 | 0.814 | 0.715 | 0.793 | 0.892 | 0.687 | | | | | | |
| G_T | 0.971 | 0.854 | 0.831 | 0.875 | 0.842 | 0.860 | 0.615 | 0.882 | 0.959 | 0.789 | 0.845 | | | | | |
| T_A | 0.738 | 0.590 | 0.792 | 0.765 | 0.928 | 0.822 | 0.840 | 0.815 | 0.731 | 0.730 | 0.756 | 0.702 | | | | |
| T_C | 0.753 | 0.762 | 0.685 | 0.747 | 0.859 | 0.909 | 0.788 | 0.896 | 0.710 | 0.732 | 0.698 | 0.754 | 0.859 | | | |
| T_G | 0.696 | 0.640 | 0.789 | 0.718 | 0.882 | 0.842 | 0.886 | 0.830 | 0.710 | 0.639 | 0.752 | 0.671 | 0.883 | 0.917 | | |
| T_T | 0.778 | 0.728 | 0.729 | 0.831 | 0.919 | 0.941 | 0.796 | 0.924 | 0.787 | 0.816 | 0.818 | 0.765 | 0.903 | 0.931 | 0.903 | |
| Mean | 0.928 | 0.831 | 0.879 | 0.924 | 0.965 | 0.961 | 0.800 | 0.957 | 0.928 | 0.834 | 0.893 | 0.914 | 0.891 | 0.904 | 0.884 | 0.942 |

**d**

Base Pair Shear

| Nearest Base Pairs | AT | AC | AG | AA | GT | GC | GG | GA | CT | CC | CG | CA | TT | TC | TG | TA |
|---|---|---|---|---|---|---|---|---|---|---|---|---|---|---|---|---|
| A_A | -2.280 | -0.473 | 2.433 | -0.121 | -3.192 | -2.256 | 0.073 | -2.650 | -2.444 | -0.246 | -6.390 | -5.147 | 0.075 | -2.868 | -0.077 | -2.283 |
| A_C | -1.706 | -0.727 | 2.096 | -0.157 | -3.965 | -2.776 | 0.048 | -3.207 | -2.460 | -0.214 | -6.498 | -0.054 | 0.101 | -3.998 | -0.982 | -5.700 |
| A_G | -0.151 | 1.063 | 2.393 | -0.102 | -2.209 | -2.720 | 0.060 | -2.309 | -2.448 | -0.190 | -6.308 | -1.141 | 0.114 | -1.631 | -0.109 | 4.868 |
| A_T | -2.723 | 0.349 | 0.682 | -0.118 | -4.363 | -4.748 | 0.020 | -3.739 | -2.409 | -0.192 | -6.583 | -2.491 | 0.089 | 6.933 | -1.336 | -5.024 |
| C_A | -1.662 | 0.653 | 2.394 | -0.109 | -2.925 | -1.349 | 0.068 | -3.005 | -2.456 | -0.227 | -6.112 | -1.978 | 0.098 | -1.601 | 0.343 | -4.031 |
| C_C | -2.882 | -0.292 | 2.368 | -0.165 | -4.546 | -3.779 | 0.062 | -3.331 | -2.454 | -0.230 | -6.287 | -2.114 | 0.122 | -3.266 | -0.700 | -5.063 |
| C_G | -0.293 | 0.936 | 1.861 | -0.120 | -2.719 | -1.826 | 0.093 | -2.869 | -2.475 | -0.194 | -5.984 | -2.942 | 0.137 | -2.680 | -0.086 | -2.766 |
| C_T | -2.347 | 1.816 | 2.329 | -0.125 | -4.178 | -3.176 | 0.035 | -3.185 | -2.410 | -0.208 | -6.252 | -1.853 | 0.127 | -0.837 | -0.239 | -4.938 |
| G_A | -1.304 | 0.808 | 0.449 | -0.085 | -4.180 | -1.213 | 0.085 | -2.569 | -2.435 | -0.217 | -4.798 | -3.238 | 0.093 | -2.423 | -0.105 | -4.407 |
| G_C | -2.735 | -1.073 | -2.839 | -0.153 | -4.323 | -2.567 | 0.054 | -3.438 | -2.500 | -0.209 | -6.024 | -5.075 | 0.125 | -1.851 | -3.646 | -3.142 |
| G_G | -0.429 | 3.528 | 2.421 | -0.112 | -4.168 | -0.270 | 0.115 | -1.712 | -2.443 | -0.184 | -4.569 | -4.932 | 0.141 | -2.366 | -0.142 | 1.139 |
| G_T | -1.828 | -0.618 | 2.342 | -0.112 | -4.592 | -0.773 | 0.055 | -2.588 | -2.422 | -0.179 | -6.297 | -2.483 | 0.112 | -1.320 | -3.338 | -5.649 |
| T_A | -1.060 | -0.032 | 0.751 | -0.091 | -2.749 | -0.966 | 0.089 | -3.035 | -2.503 | -0.210 | -5.948 | -0.427 | 0.100 | 1.315 | -0.294 | -1.149 |
| T_C | -2.723 | 1.286 | 2.398 | -0.158 | -4.633 | -2.602 | 0.078 | -3.902 | -2.447 | -0.217 | -6.075 | -0.084 | 0.092 | 0.551 | -1.846 | -4.665 |
| T_G | 0.534 | 3.268 | 2.405 | -0.103 | -3.397 | -3.764 | 0.080 | -1.563 | -2.466 | -0.199 | -5.768 | -5.190 | 0.143 | 1.446 | 0.278 | 2.053 |
| T_T | -2.028 | -0.181 | 0.894 | -0.101 | -3.823 | -2.820 | 0.033 | -3.946 | -2.421 | -0.201 | -6.010 | -2.653 | 0.107 | -0.423 | -1.304 | -4.432 |
| Mean | -1.601 | 0.644 | 1.586 | -0.121 | -3.748 | -2.350 | 0.066 | -2.941 | -2.450 | -0.207 | -5.994 | -2.613 | 0.111 | -0.939 | -0.849 | -2.824 |

Base Pair Type

| Nearest Base Pairs | A_A | A_C | A_G | A_T | C_A | C_C | C_G | C_T | G_A | G_C | G_G | G_T | T_A | T_C | T_G | T_T |
|---|---|---|---|---|---|---|---|---|---|---|---|---|---|---|---|---|
| A_C | 0.736 | | | | | | | | | | | | | | | |
| A_G | 0.643 | 0.391 | | | | | | | | | | | | | | |
| A_T | 0.478 | 0.455 | 0.263 | | | | | | | | | | | | | |
| C_A | 0.888 | 0.905 | 0.506 | 0.619 | | | | | | | | | | | | |
| C_C | 0.863 | 0.951 | 0.476 | 0.574 | 0.945 | | | | | | | | | | | |
| C_G | 0.930 | 0.850 | 0.635 | 0.492 | 0.949 | 0.900 | | | | | | | | | | |
| C_T | 0.818 | 0.880 | 0.464 | 0.737 | 0.957 | 0.950 | 0.890 | | | | | | | | | |
| G_A | 0.857 | 0.838 | 0.376 | 0.536 | 0.926 | 0.896 | 0.920 | 0.904 | | | | | | | | |
| G_C | 0.713 | 0.536 | 0.415 | 0.586 | 0.640 | 0.641 | 0.689 | 0.656 | 0.746 | | | | | | | |
| G_G | 0.821 | 0.464 | 0.729 | 0.297 | 0.679 | 0.610 | 0.802 | 0.643 | 0.736 | 0.615 | | | | | | |
| G_T | 0.765 | 0.854 | 0.318 | 0.631 | 0.881 | 0.865 | 0.813 | 0.866 | 0.857 | 0.714 | 0.565 | | | | | |
| T_A | 0.686 | 0.678 | 0.654 | 0.781 | 0.820 | 0.713 | 0.756 | 0.804 | 0.685 | 0.647 | 0.564 | 0.734 | | | | |
| T_C | 0.673 | 0.841 | 0.426 | 0.798 | 0.881 | 0.877 | 0.767 | 0.947 | 0.787 | 0.616 | 0.513 | 0.878 | 0.854 | | | |
| T_G | 0.748 | 0.357 | 0.790 | 0.594 | 0.611 | 0.548 | 0.721 | 0.649 | 0.589 | 0.601 | 0.847 | 0.468 | 0.675 | 0.547 | | |
| T_T | 0.827 | 0.843 | 0.459 | 0.804 | 0.935 | 0.915 | 0.877 | 0.957 | 0.889 | 0.783 | 0.601 | 0.897 | 0.862 | 0.933 | 0.648 | |
| Mean | 0.904 | 0.837 | 0.628 | 0.718 | 0.955 | 0.925 | 0.943 | 0.959 | 0.907 | 0.768 | 0.764 | 0.879 | 0.873 | 0.904 | 0.769 | 0.966 |

Nearest Base Pairs

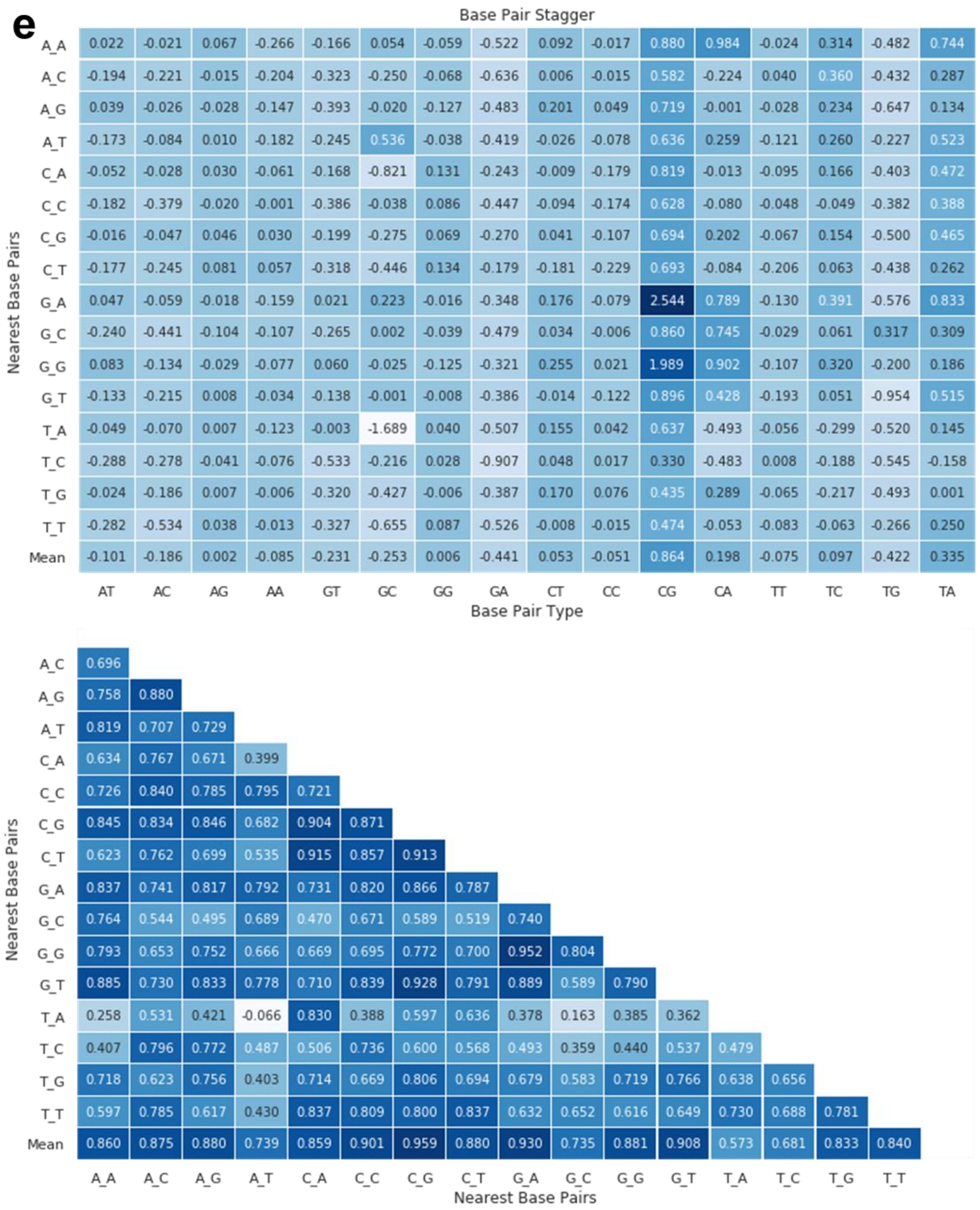


**Fig. S1 Analysis of molecular interaction features.** Features of hydrogen bonds (**a**), free energy contribution of RNA nucleotide (**b**), distance between RNA@P and DNA@P atoms (**c**), base pair shear (**d**) and base pair stagger (**e**) calculated from feature analysis (left plots). Pearson's correlations of the features derived from different nearest base pairs, which are the Pearson's correlations of different rows in left plots, are shown in the right plots.

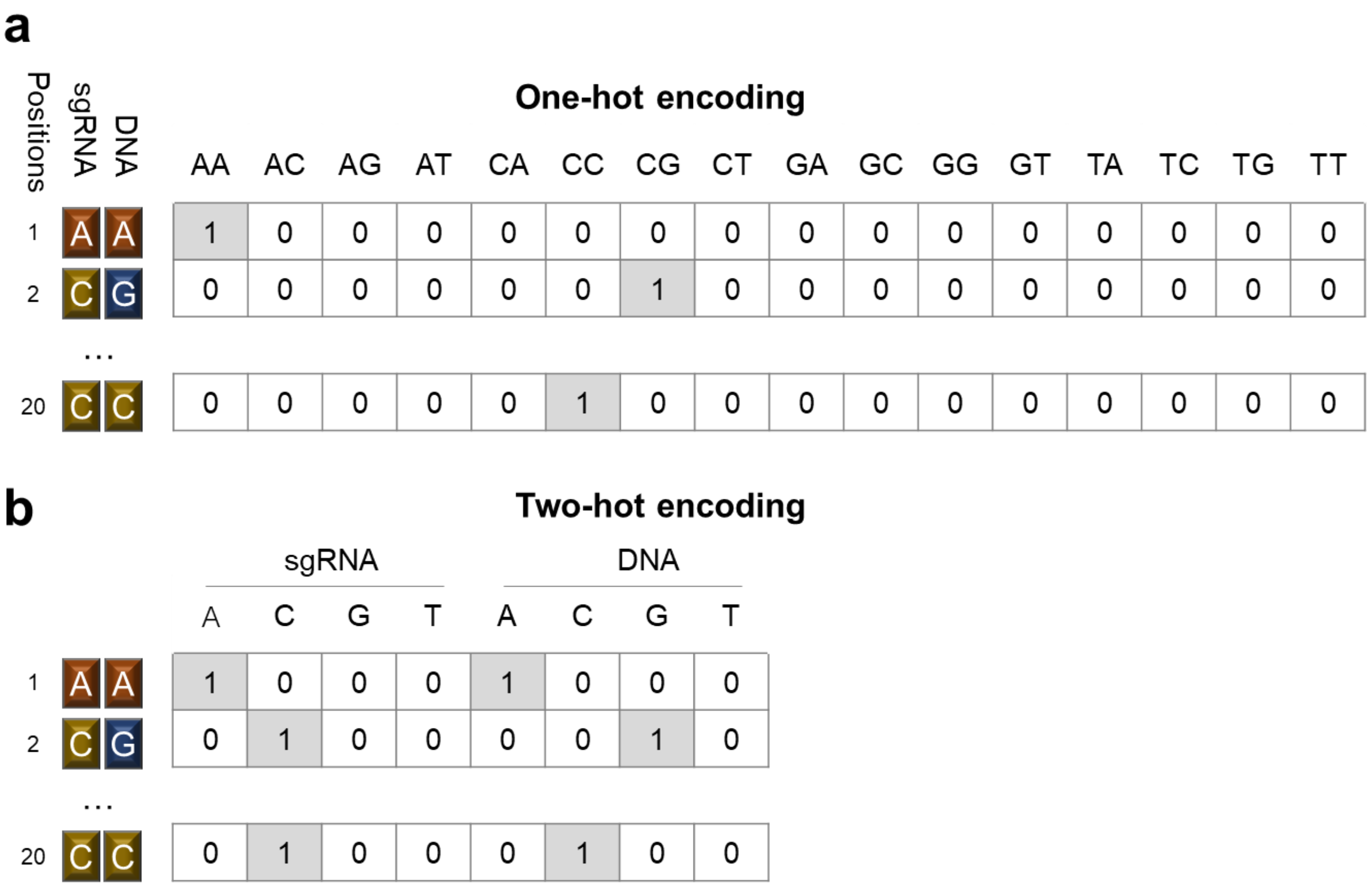


**Fig. S2 Onehot and twohot encoding methods. a**, Onehot encoding, each position of the sgRNA-DNA sequence pair was encoded by a 16-bit binary vector. **b**, Twohot encoding, each position of sgRNA and DNA sequence was encoded by a 4-bit binary vector, resulting to a 8-bit vector for the base pair.

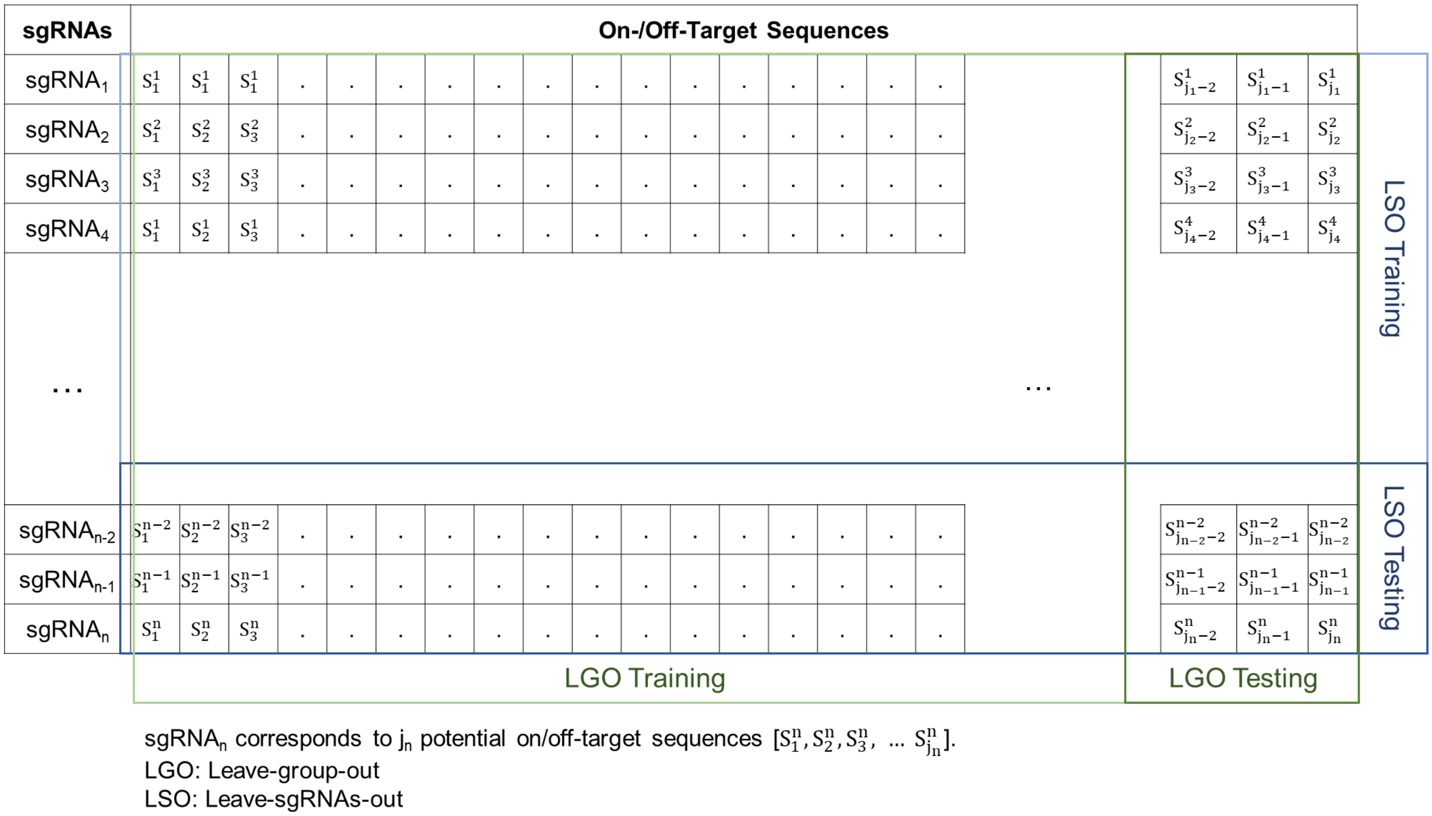


**Fig. S3 Data splitting of the leave-group-out (LGO) and leave-sgRNAs-out (LSO) tests.** A sgRNA ($sgRNA_n$) leads to $j_n$ potential on/off-target sequences $[S_1^n, S_2^n, S_3^n, \ \ldots \ S_{j_n}^n]$. An input instance is a pair of sgRNA and the on-/off-target sequence (e.g. $sgRNA_n$ and $S_{j_n}^n$). LGO test randomly held out 1/5 of the inputs as testing data, so that the training and testing contain all sgRNAs and different sequences of their corresponding on-/off-target sites. LSO test randomly held out 1/5 of the sgRNAs and the corresponding on-/off-target sequences as testing data, so that training and testing datasets contained different sgRNAs and off-target sequences.

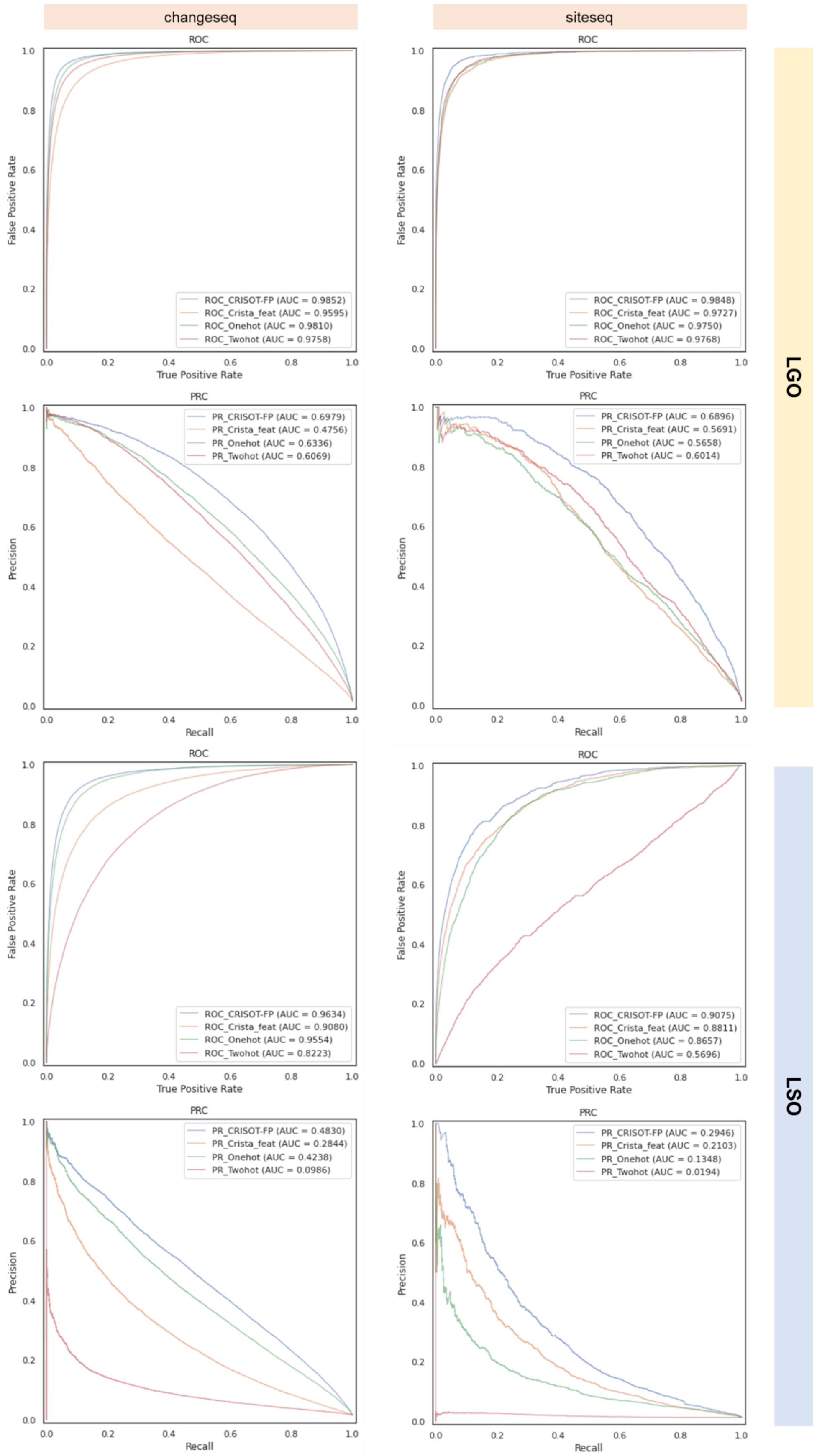


**Fig. S4 ROC and PR curves in the leave-ones-out evaluations.** The upper and lower plots are leave-groups-out (LGO) and leave-sgRNAs-out (LSO) evaluations. The models are trained using XGBoost classifier algorithm, randomly holding out 1/5 of inputs (LGO) and 1/5 of sgRNAs (LSO) as testing sets.

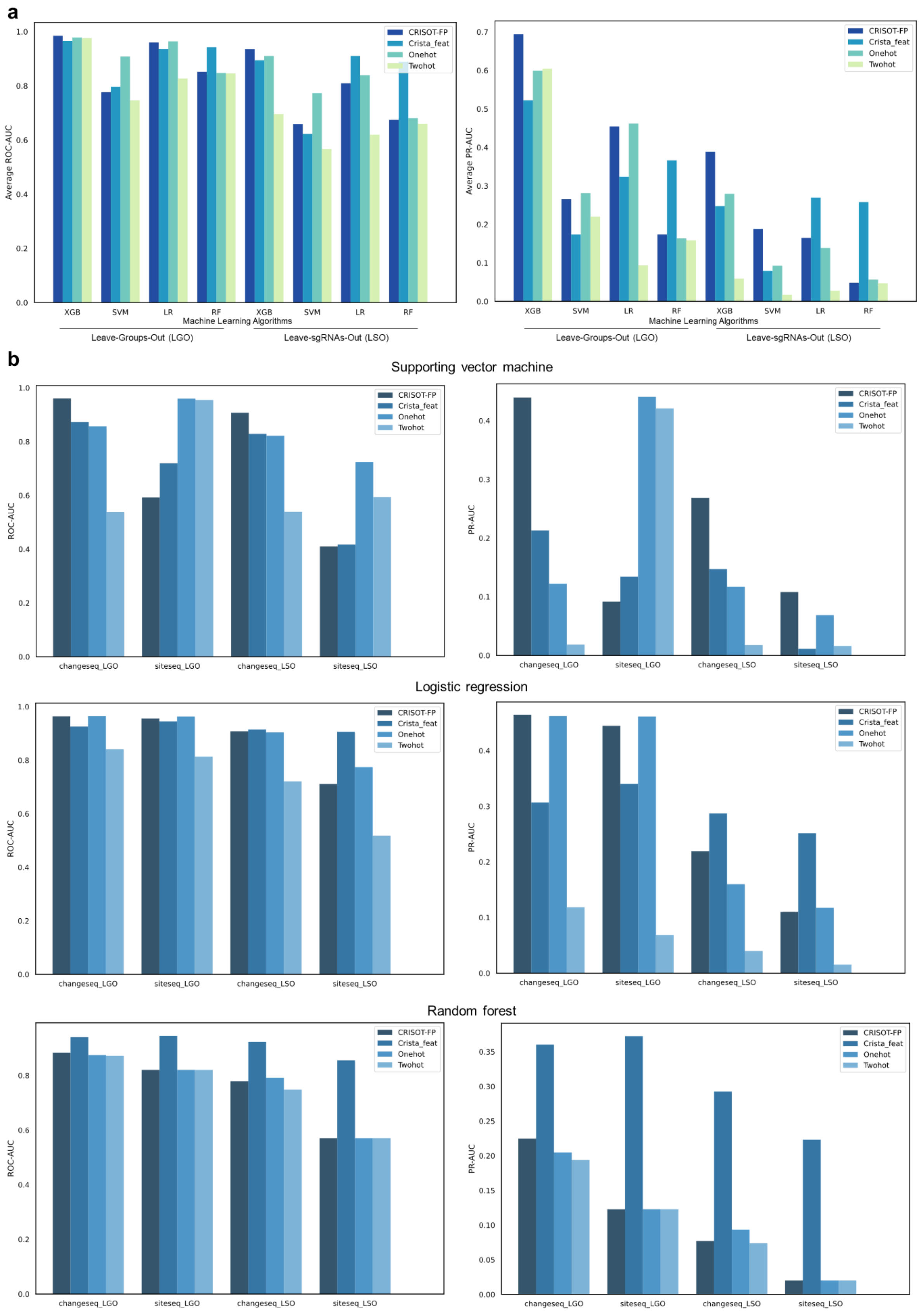


**Fig. S5 ROC-AUC and PR-AUC analysis in the leave-ones-out evaluations using various machine learning algorithms.** Both leave-groups-out (LGO) and leave-sgRNAs-out (LSO) evaluations are shown. The models are trained using XGBoost (XGB), supporting vector machine (SVM), logistic regression (LR) and random forest (RF) algorithms, randomly holding out 1/5 of inputs (LGO) and 1/5 of sgRNAs (LSO) as testing sets. Results of the means (**a**) of the different benchmark datasets and the independent performances (**b**) in different benchmark datasets are shown. Crista_feat win better performances in some schemes, because it contains some features, e.g. the number of mismatches, that are easy to interpret.

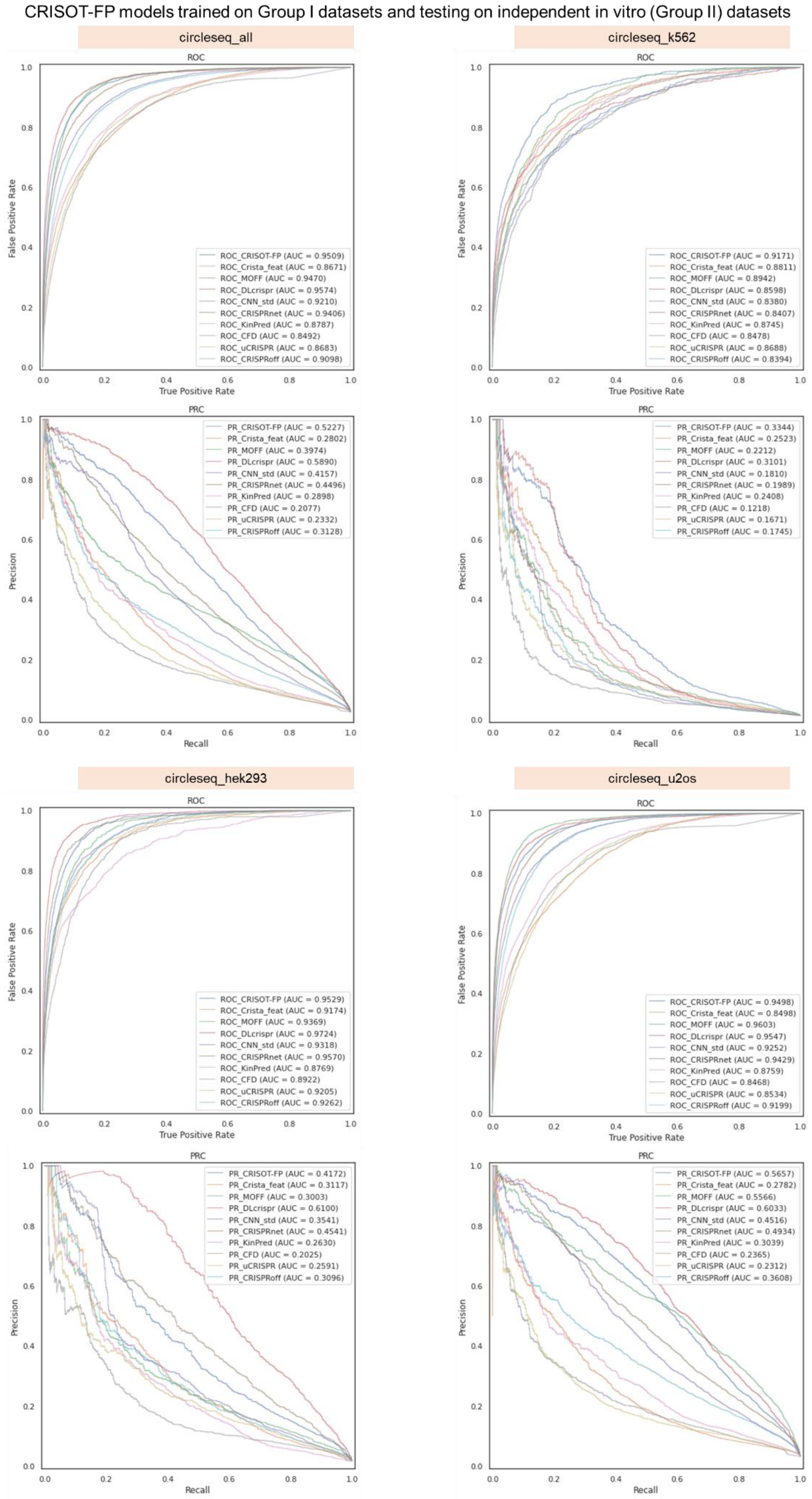


**Fig. S6 Results of CRISOT-FP models and existing off-target prediction methods in predicting independent *in vitro* off-target datasets.** Models are XGBoost models developed by leave-ones-out training using the Group I datasets.

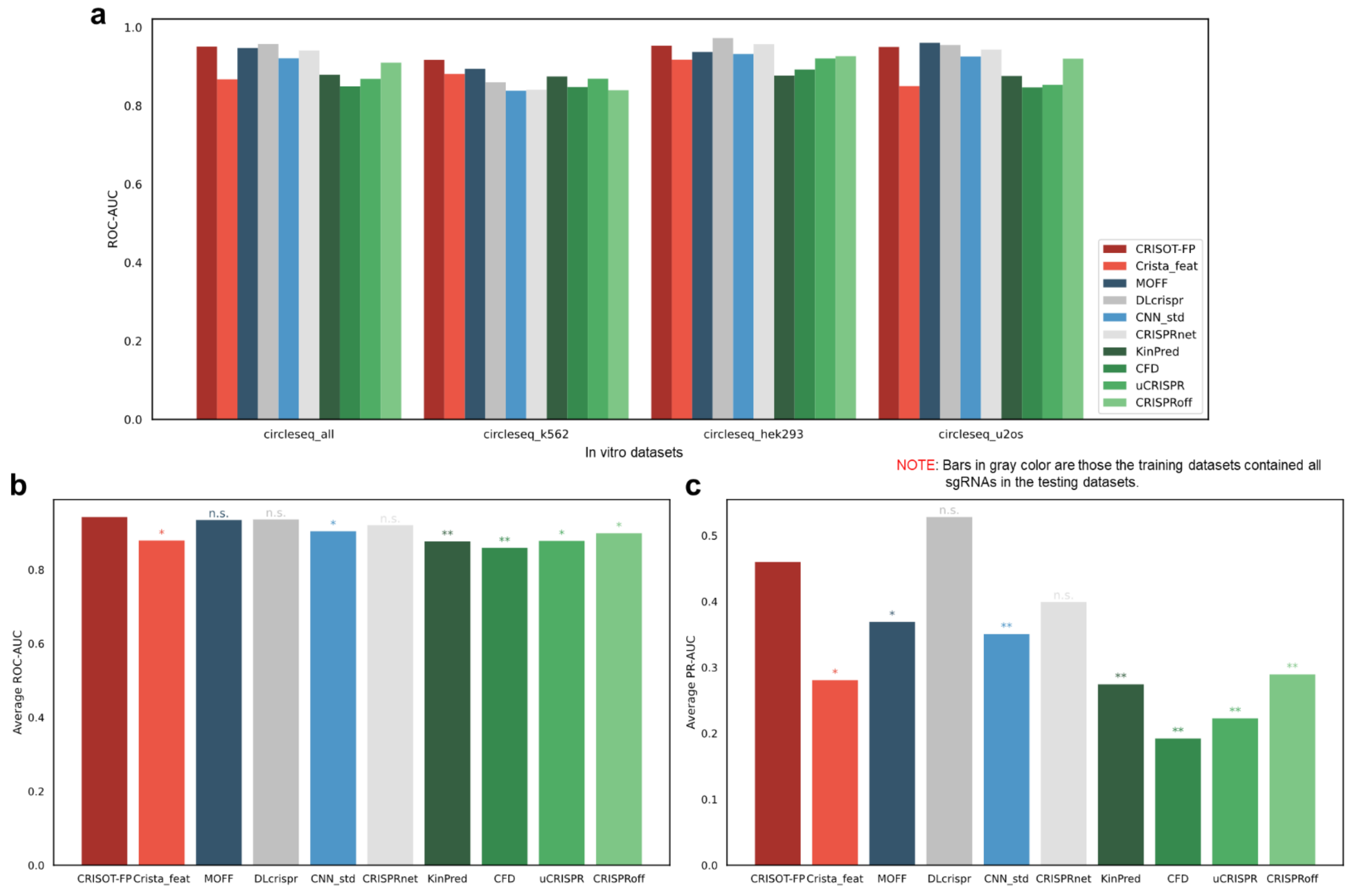


**Fig. S7 Comparison of CRISOT-FP models with off-target prediction methods in predicting independent *in vitro* off-target datasets. a**, The ROC-AUC results**.** The blue and green bars represent learning-based and hypothesis-driven methods, respectively. Bars in gray color are those the training datasets contained all sgRNAs in the testing datasets. Bars with slash hatch are presented by excluding the overlapped sgRNAs. The overlapped sgRNAs are summarized in Supplementary Data 3. The CRISOT-FP models are trained on the Group I datasets, which are independent to the testing datasets. **b, c,** One-sided paired t-test of the PR-AUC and ROC-AUC, respectively. n.s.: not significant, *: p<0.05, **: p<0.01, ***: p<0.001, n=4. The p-values for the ROC-AUC results are 0.015, 0.175, 0.364, 0.033, 0.154, 0.002, 0.002, 0.011 and 0.016, respectively. The p-values for the PR-AUC results are 0.019, 0.023, 0.883, 0.005, 0.094, 0.008, 0.002, 0.006 and 0.003, respectively.

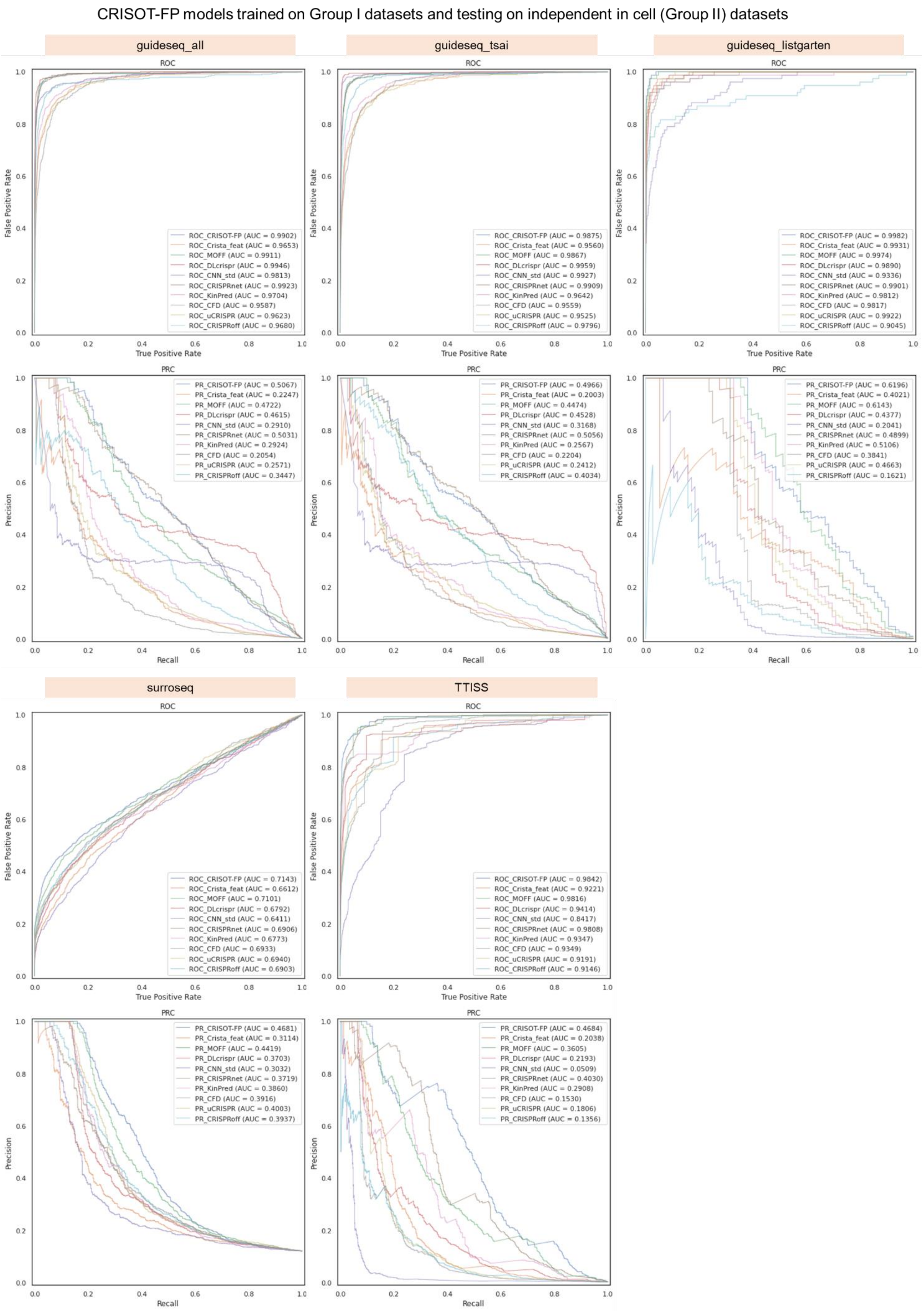


**Fig. S8 Results of CRISOT-FP models and existing off-target prediction methods in predicting independent in cell off-target datasets.** Models are XGBoost models developed by leave-ones-out training using the Group I datasets.

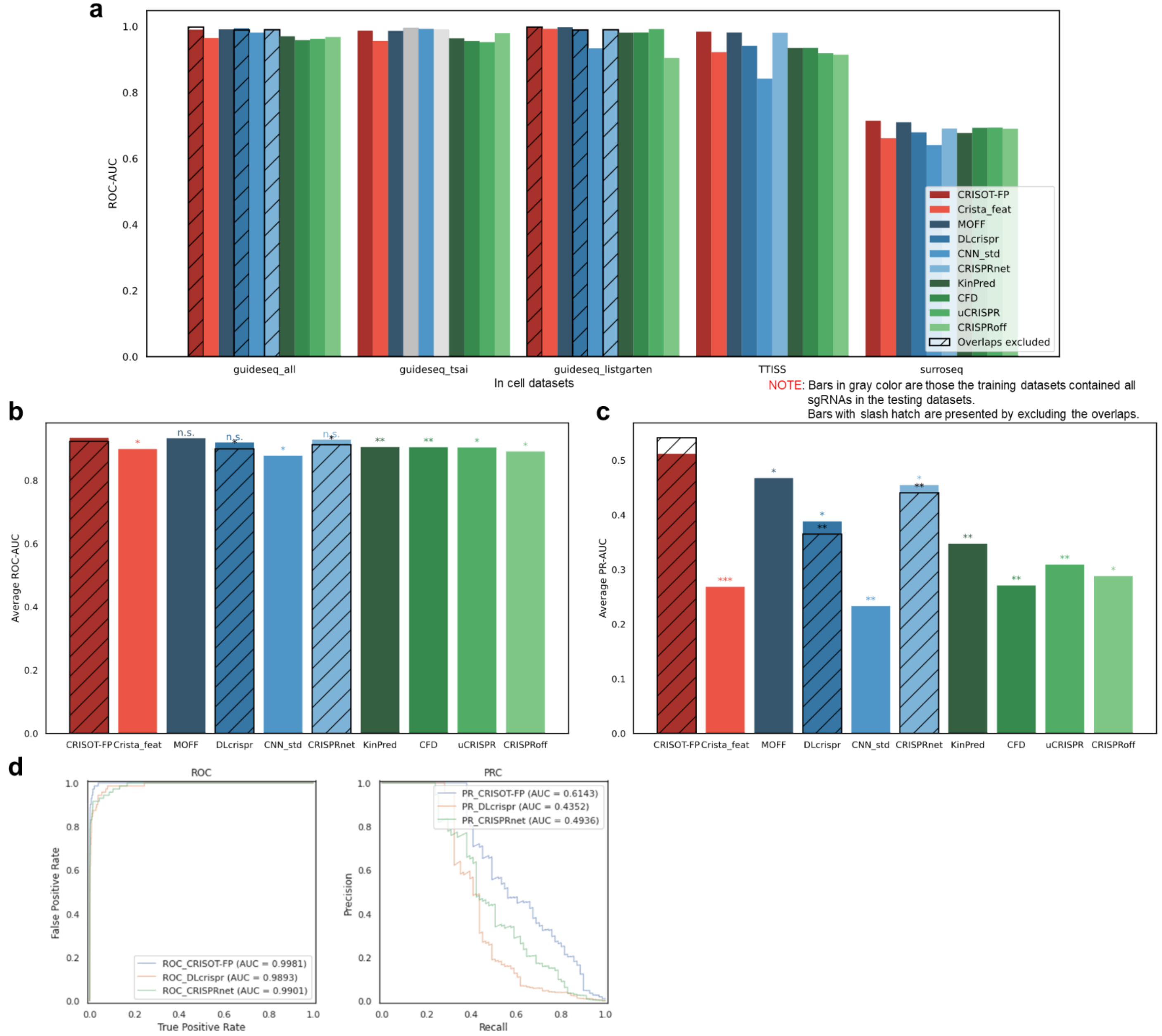


**Fig. S9 Comparison of CRISOT-FP with state-of-the-art off-target prediction methods in predicting independent in cell datasets. a**, The ROC-AUC results. The blue and green bars represent learning-based and hypothesis-driven methods, respectively. For the compared methods whose training datasets contained sgRNAs in the testing dataset, additional bars in slash hatch were shown. Bars in gray color are those the training datasets contained all sgRNAs in the testing datasets. Bars with slash hatch are presented by excluding the overlapped sgRNAs. Of note, for fair comparisons, the same sgRNAs were removed from the testing datasets for CRISOT-FP. The overlapped sgRNAs are summarized in Supplementary Data 3. The CRISOT-FP models are trained on the Group I datasets, which are independent to the testing datasets. **b, c,** One-sided paired t-test of the PR-AUC and ROC-AUC, respectively. n.s.: not significant, *: $p<0.05$, **: $p<0.01$, ***: $p<0.001$, n=5. Prediction results of one dataset are calculated using the models trained on the other two datasets. **d**, ROC and PR curves for the datasets that partially overlap the training datasets of the comparing models. Overlap sgRNAs are removed from the testing datasets. The p-values for the ROC-AUC results are 0.013, 0.078, 0.112, 0.048, 0.145, 0.004, 0.003, 0.017 and 0.028, respectively. The p-values for the PR-AUC results are 0.0003, 0.031, 0.019, 0.004, 0.049, 0.003, 0.003, 0.004, 0.019, respectively. The p-values excluding overlaps are 0.037 and 0.046 for ROC-AUCs and 0.005 and 0.002 for PR-AUCs.

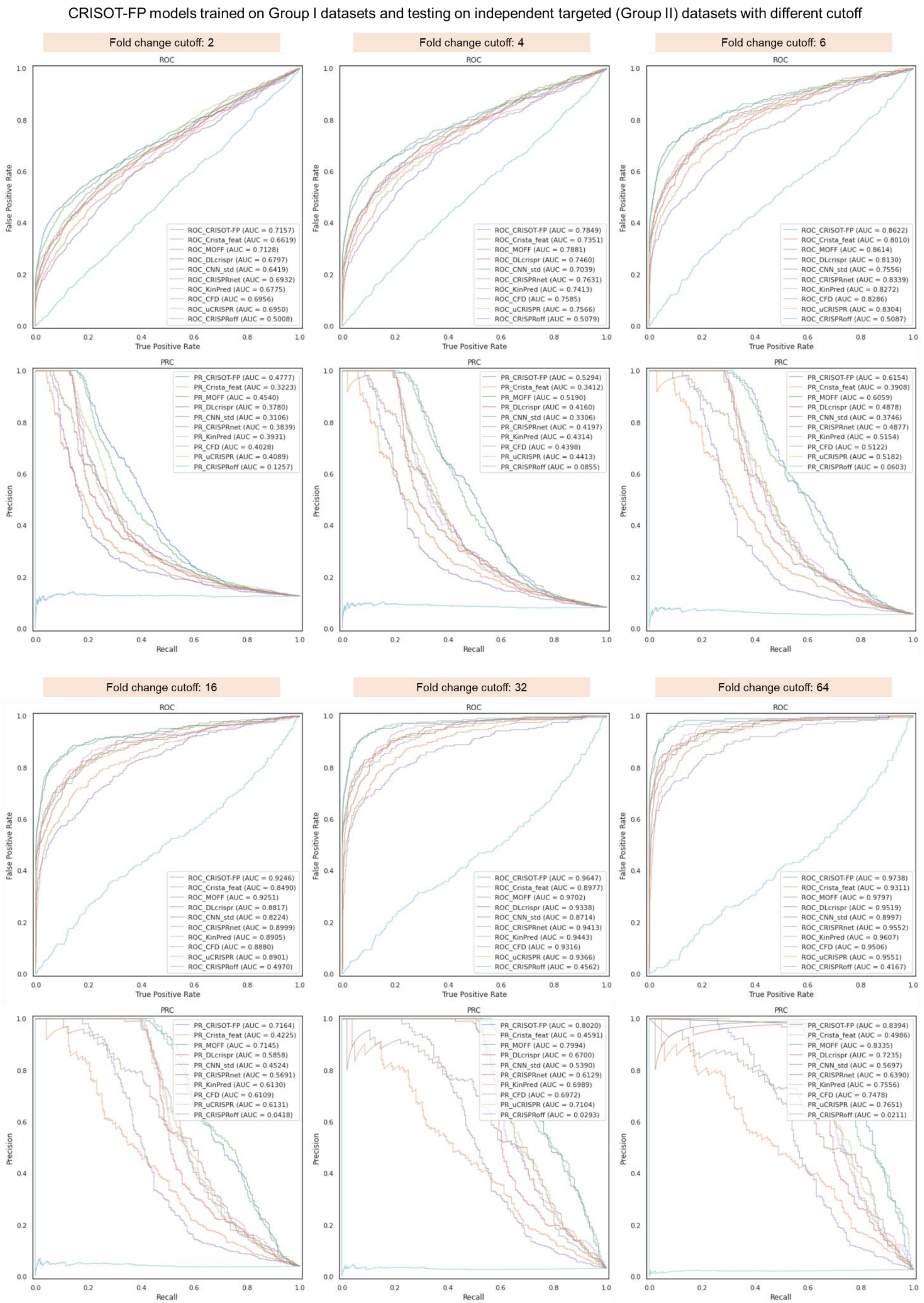


**Fig. S10 Results of CRISOT-FP models and existing off-target prediction methods in predicting independent targeted off-target datasets with different cutoff.** Models are XGBoost models developed by leave-ones-out training using the Group I datasets.

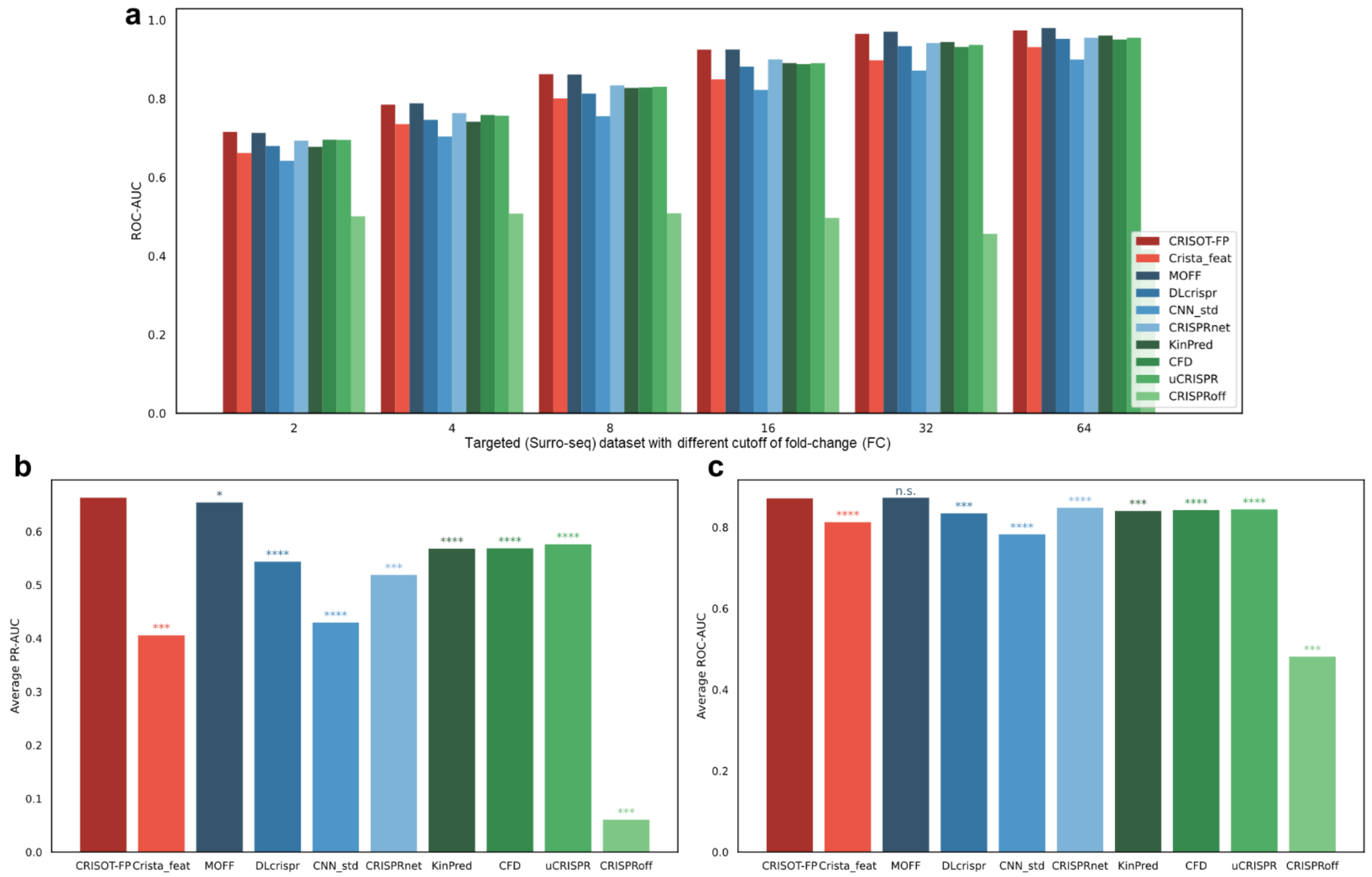


**Fig. S11 Comparison of CRISOT-FP with state-of-the-art off-target prediction methods in predicting independent targeted off-target datasets with different cutoff. a**, The ROC-AUC results. The blue and green bars represent learning-based and hypothesis-driven methods, respectively. The CRISOT-FP models are trained on the Group I datasets, which are independent to the testing datasets. **b**, **c**, One-sided paired t-test of the PR-AUC and ROC-AUC, respectively. n.s.: not significant, *: $p<0.05$, **: $p<0.01$, ***: $p<0.001$, ****: $p<0.0001$, $n=6$. The p-values for the ROC-AUC results are 0.00004, 0.875, 0.0001, 0.00001, 0.000005, 0.0006, 0.00006, 0.00006 and 0.0004, respectively. The p-values for the PR-AUC results are 0.0002, 0.020, 0.000001, 0.00002, 0.0002, 0.0000008, 0.000003, 0.000009 and 0.0002, respectively.

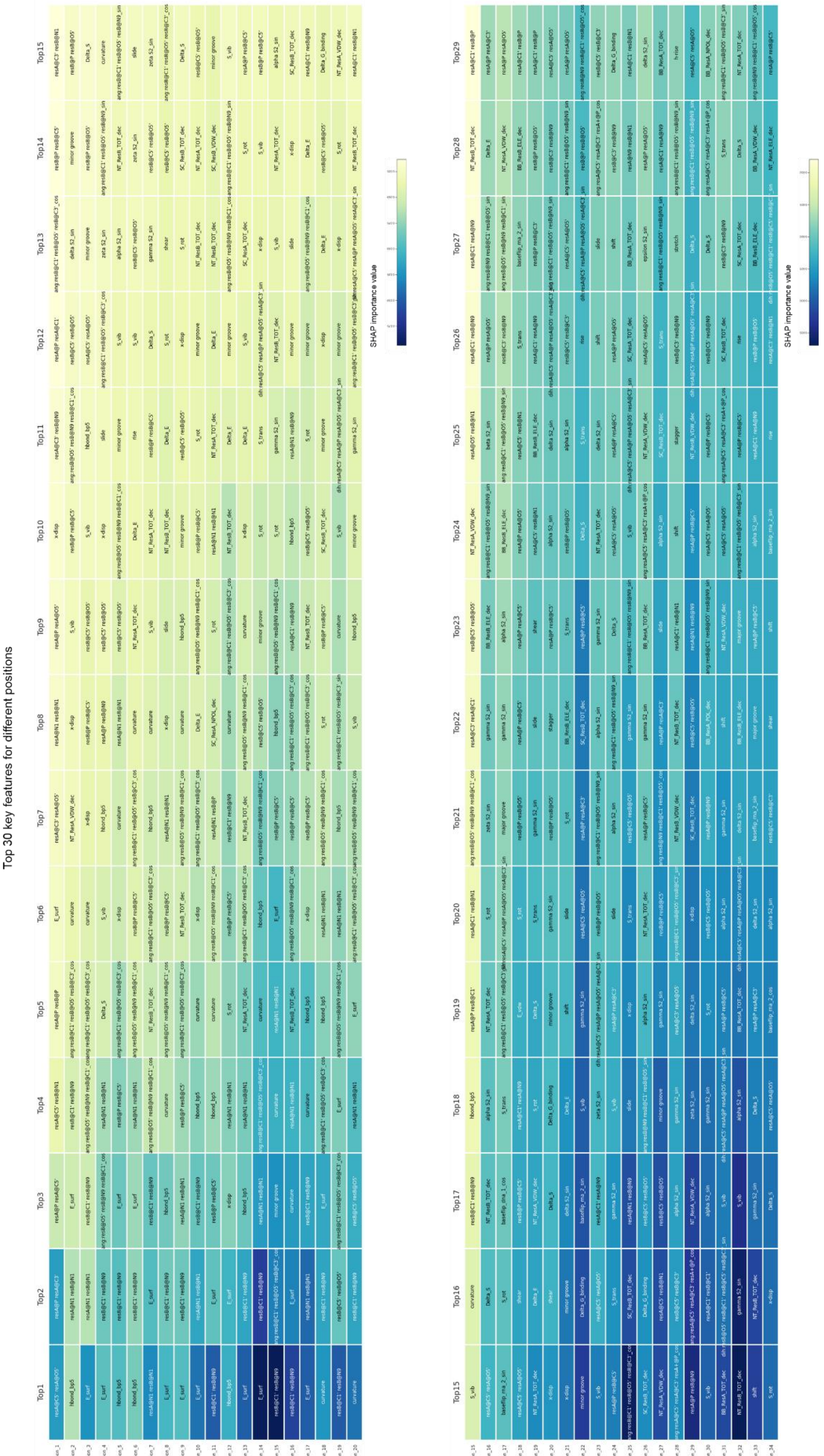


**Fig. S12 Top 30 important features of the 20 positions.** The molecular interaction features are used to develop CRISOT-Score.

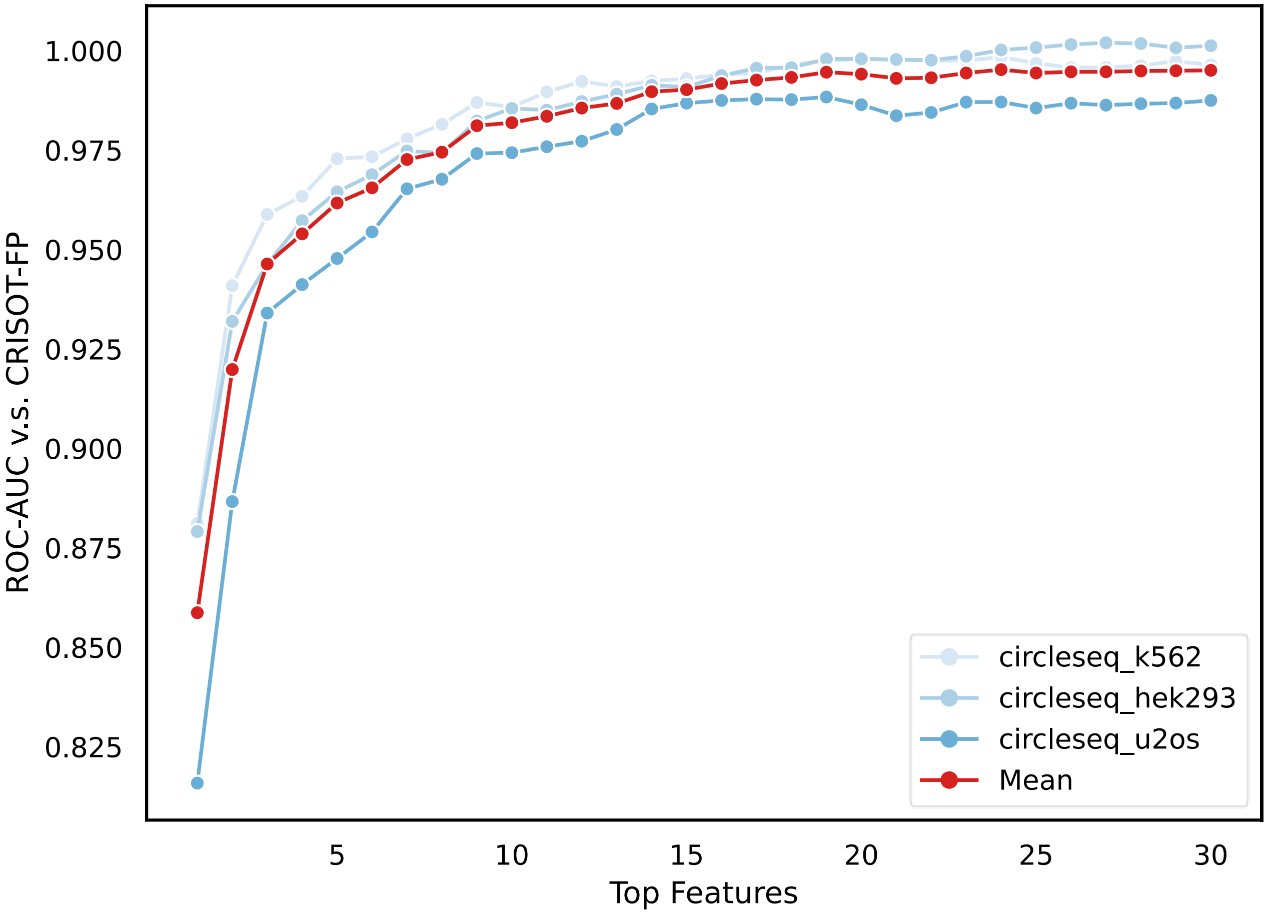


**Fig. S13 ROC-AUC Performances of CRISOT-Score developed using different numbers of key RNA-DNA molecular interaction features for each position.** Results on the different benchmark datasets are compared to the results achieved by the CRISOT-FP models.

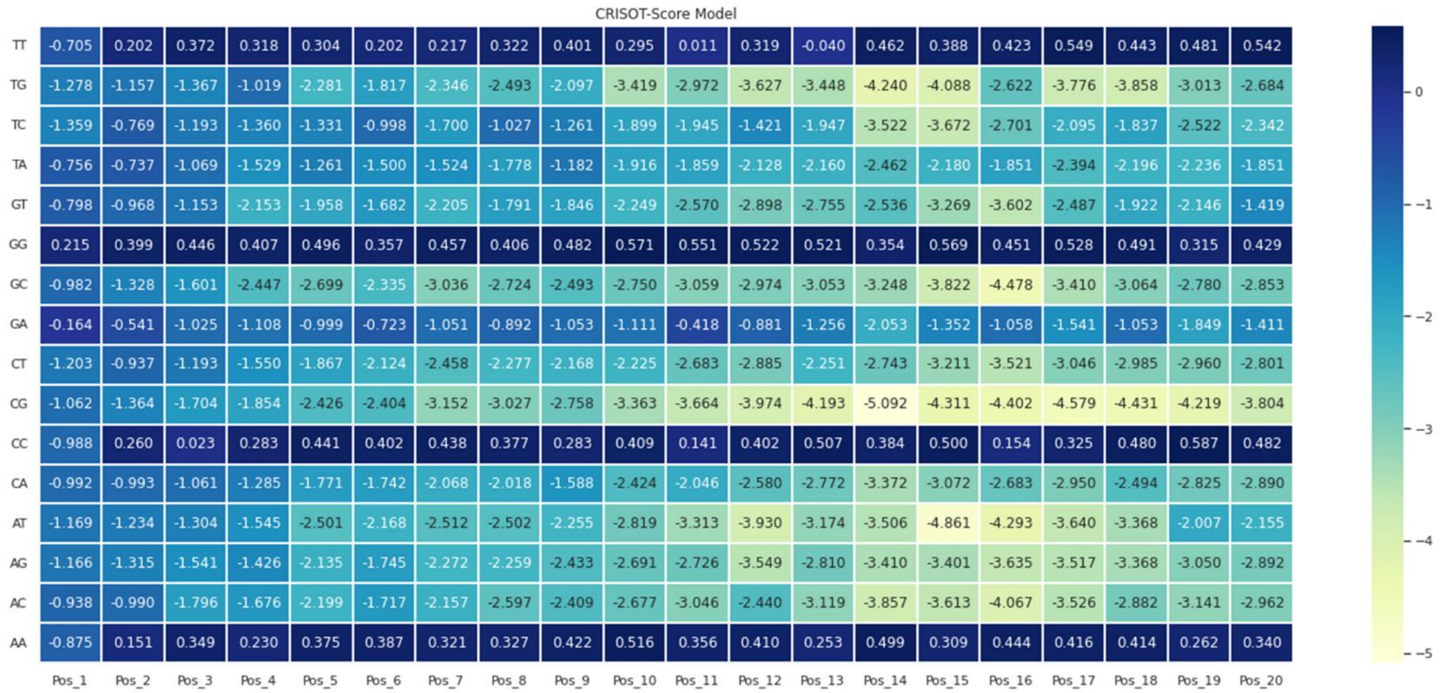


**Fig. S14 Score map of CRISOT-Score developed using the top 15 key molecular interaction features for each position.** The values are used to score a specific sgRNA-DNA. The bps are presented as sgRNA vs. off-target sequences.

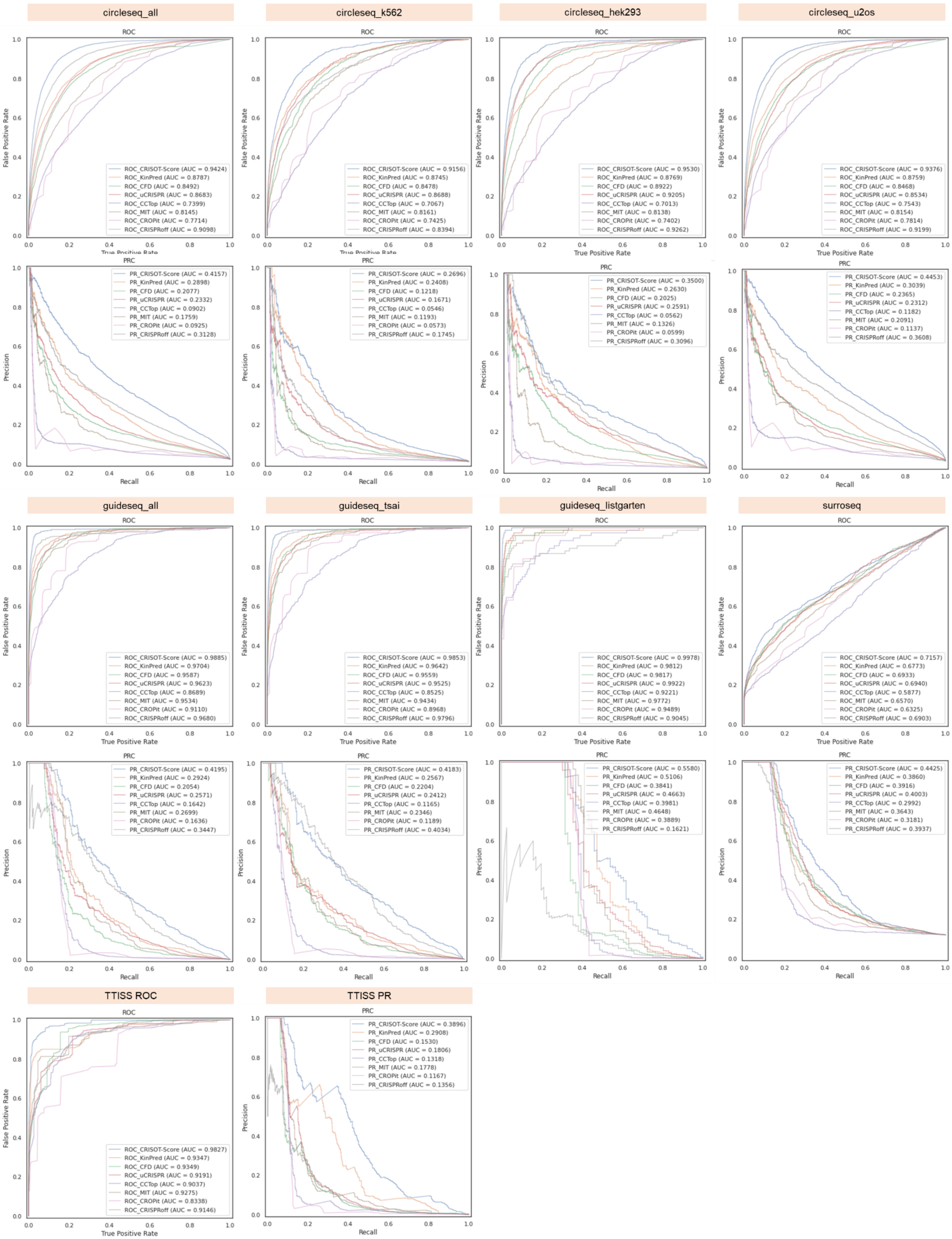


**Fig. S15 Comparison of CRISOT-Score with off-target scoring methods in predicting off-target effects.** ROC and PR curves were shown.

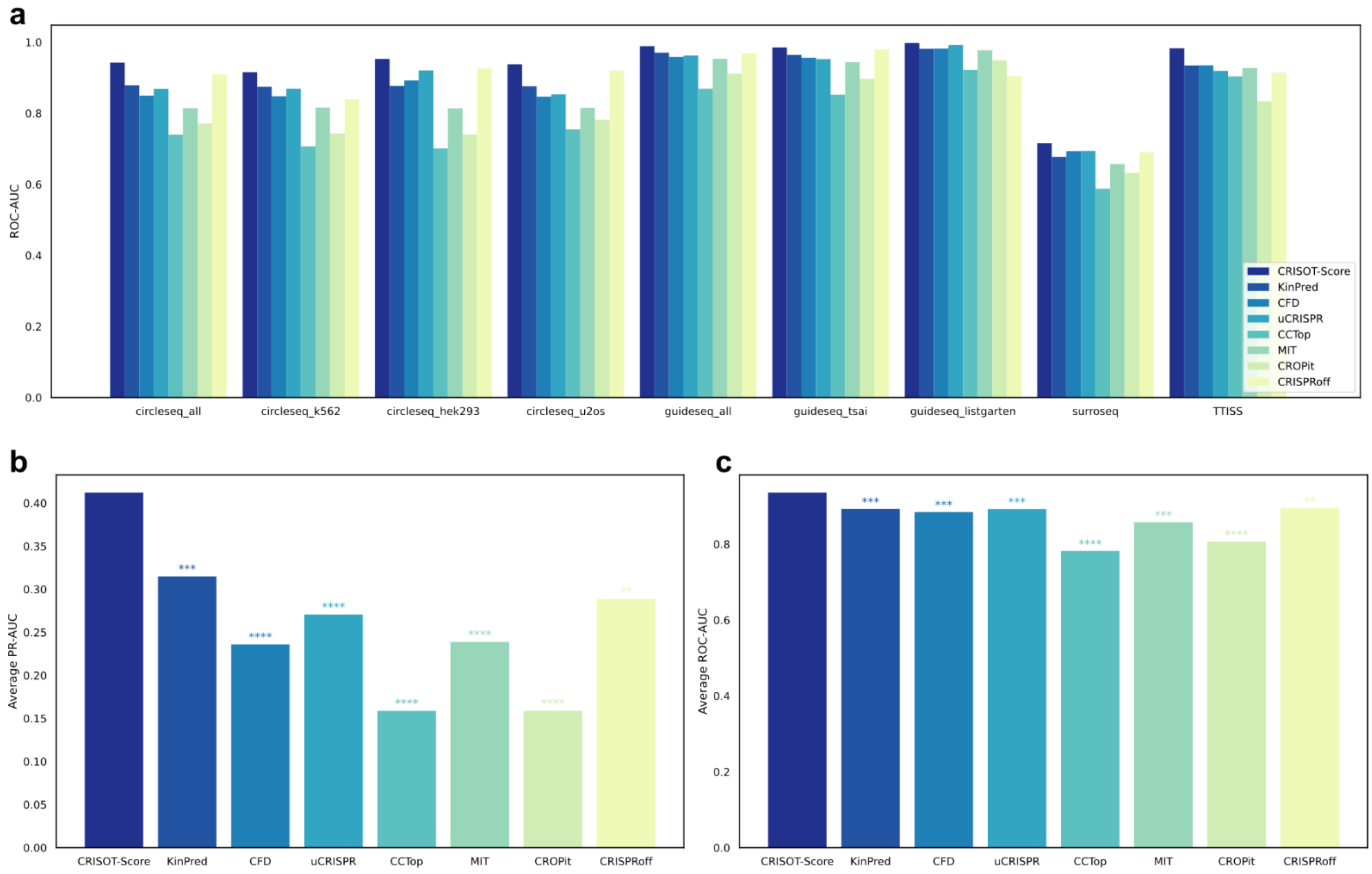


**Fig. S16 Comparison of CRISOT-Score with state-of-the-art off-target scoring methods using the Circle-seq and Group II datasets. a**, The ROC-AUC results. **b**, **c**, One-sided paired t-test of the PR-AUC and ROC-AUC, respectively. *: $p<0.05$, **: $p<0.01$, ***: $p<0.001$, ****: $p<0.0001$, $n=9$. The p-values for the ROC-AUC results are 0.0002, 0.0004, 0.0006, 0.00003, 0.0004, 0.00006 and 0.002, respectively. The p-values for the PR-AUC results are 0.0001, 0.000006, 0.00006, 0.000002, 0.00001, 0.000003 and 0.008, respectively.

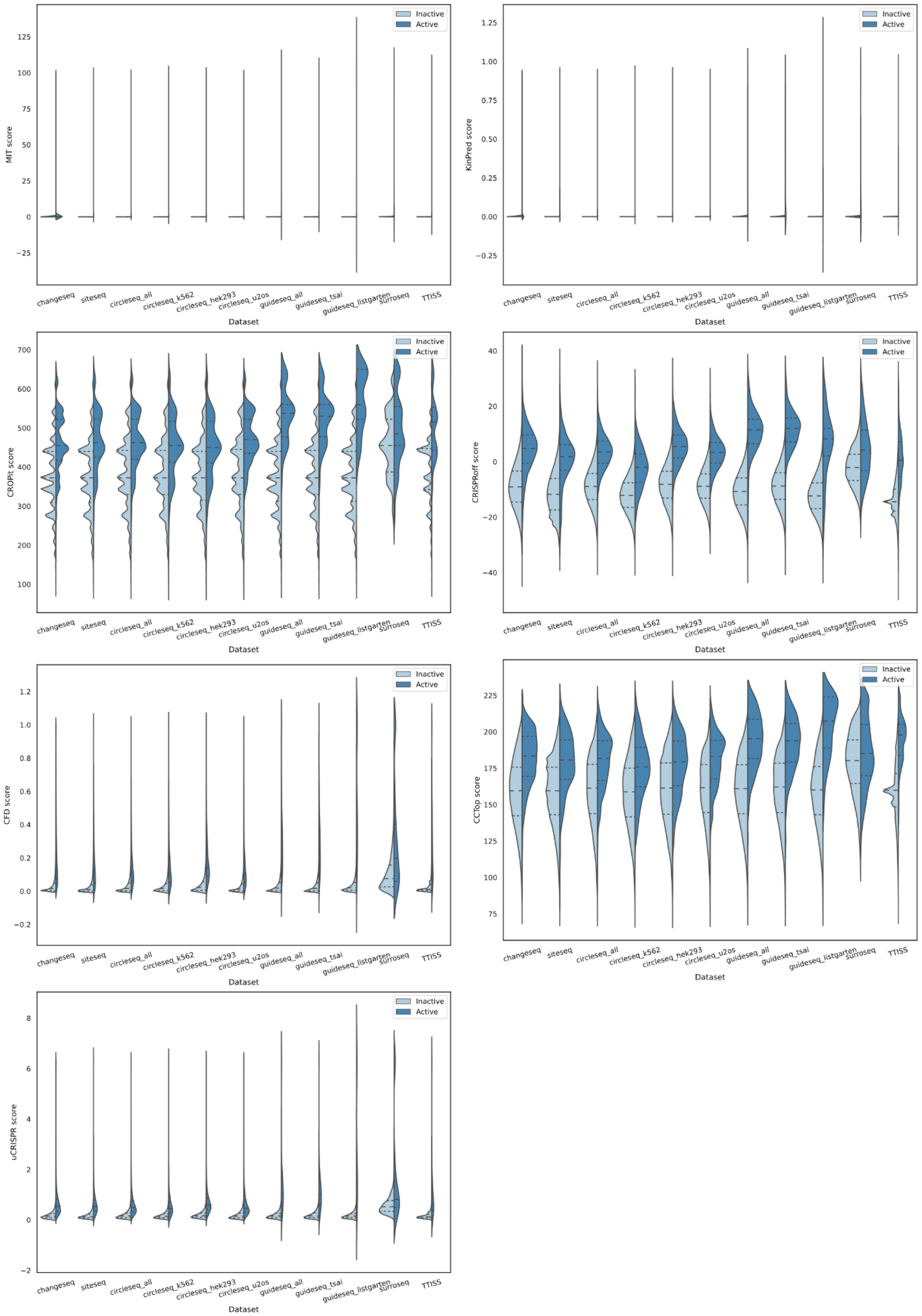


**Fig. S17 Violin plots of various off-target scores on active and inactive off-target sites of different datasets.** The numbers of active and inactive off-target sites can be found in Table 1. Among all potential off-target sites of the datasets, the experimentally validated ones were defined as active off-targets, and the rests were inactive off-targets. The three dashed lines in each violin show the quartiles of the data.

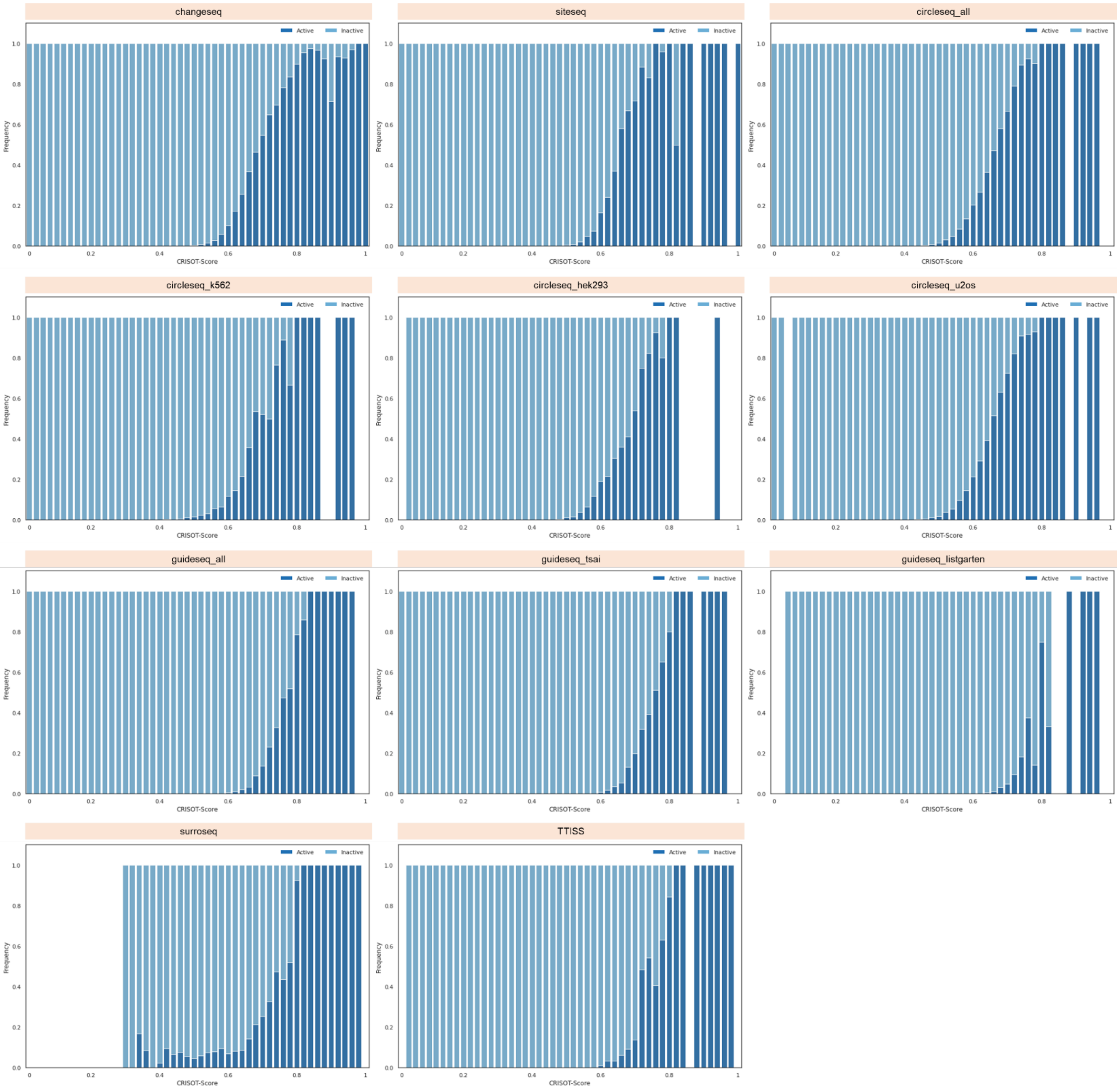


**Fig. S18 Frequencies of active and inactive off-target sites that are in different ranges of CRISOT-Score.** The frequencies of different CRISOT-Score ranges are shown. Active and inactive off-targets were shown as dark and light blue columns, respectively.

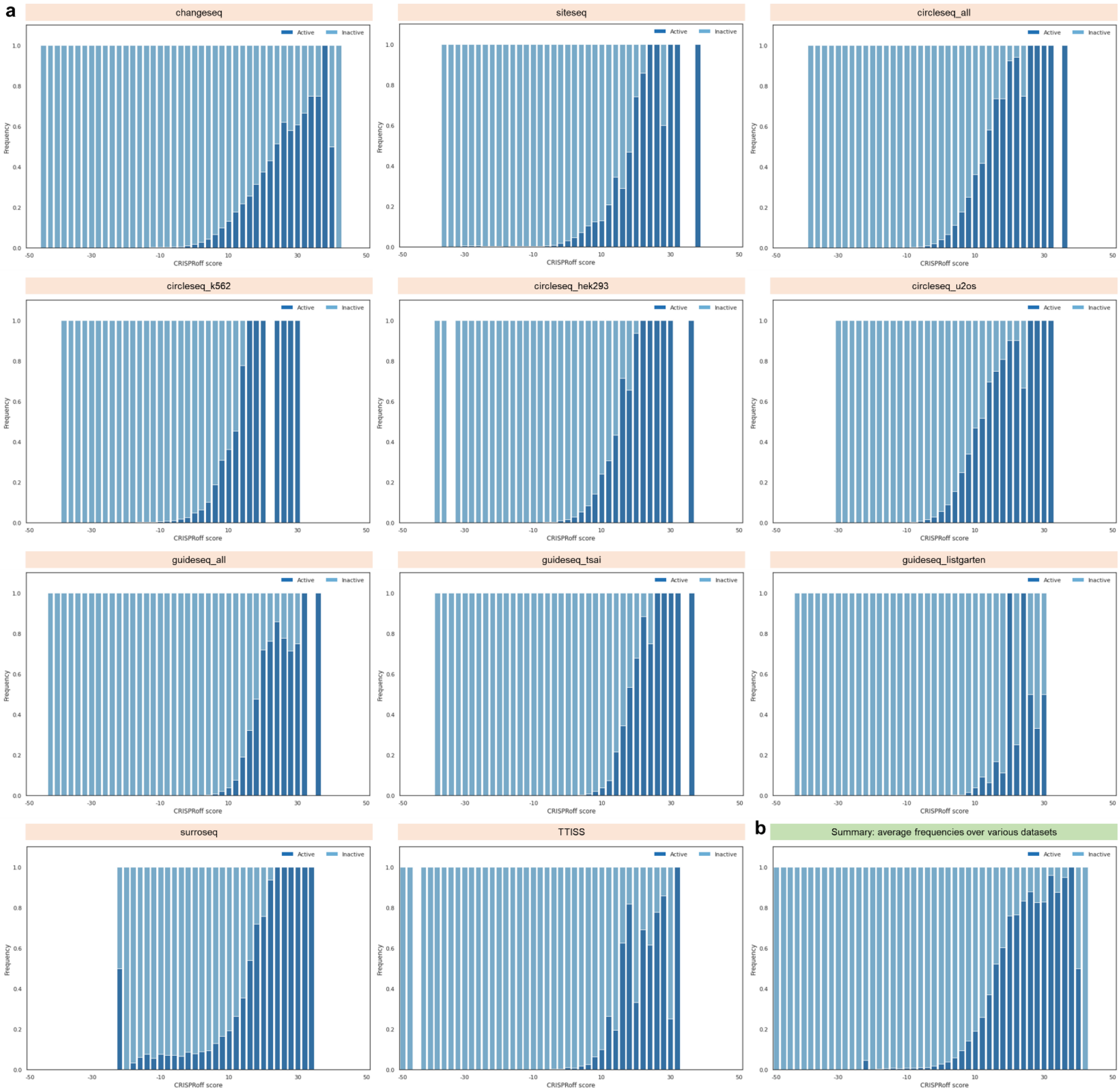


**Fig. S19 Frequencies of active and inactive off-target sites that are in different ranges of CRISPRoff score.** The frequencies of different CRISPRoff score ranges are shown. Active and inactive off-targets were shown as dark and light blue columns, respectively.

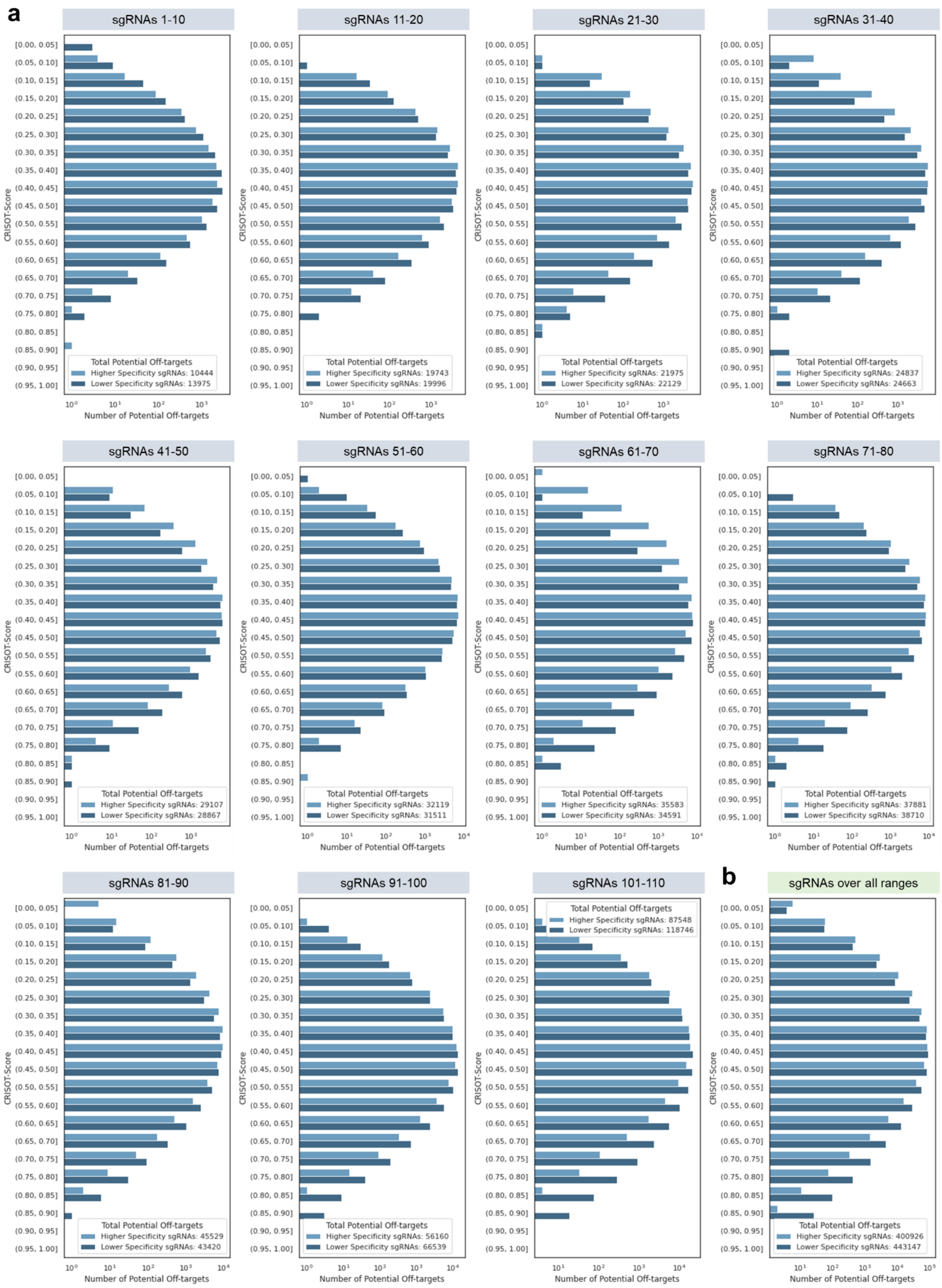


**Fig. S20. Numbers of potential off-target sites of Change-seq dataset in different CRISOT-Score ranges.** Top and bottom three sgRNAs with the least and most experimental off-target sites are selected as the higher and lower specificity sgRNAs, respectively, for each group of 10 sgRNAs.

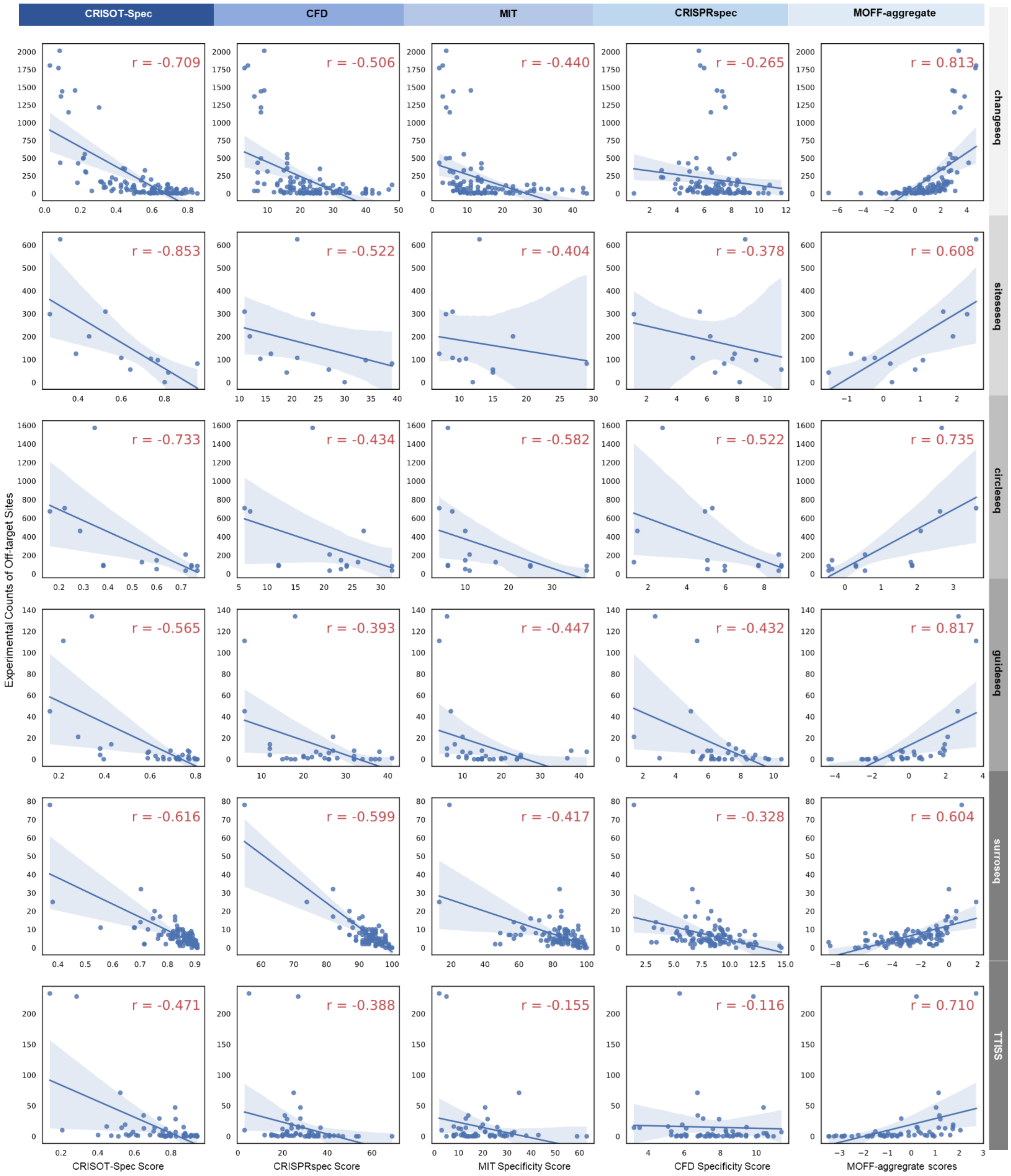


**Fig. S21. Correlations between specificity scores and the experimental counts of off-target sites reported by different experiments.** An sgRNA with a higher CRISOT-Spec score means higher specificity across the whole genome, thus stronger negative correlations between conventional specificity score and experimental off-target read fraction are expected. CRISOT-Spec is compared to three existing specificity scoring methods. The error bands represent the confidence intervals of 95% for the regression estimates.

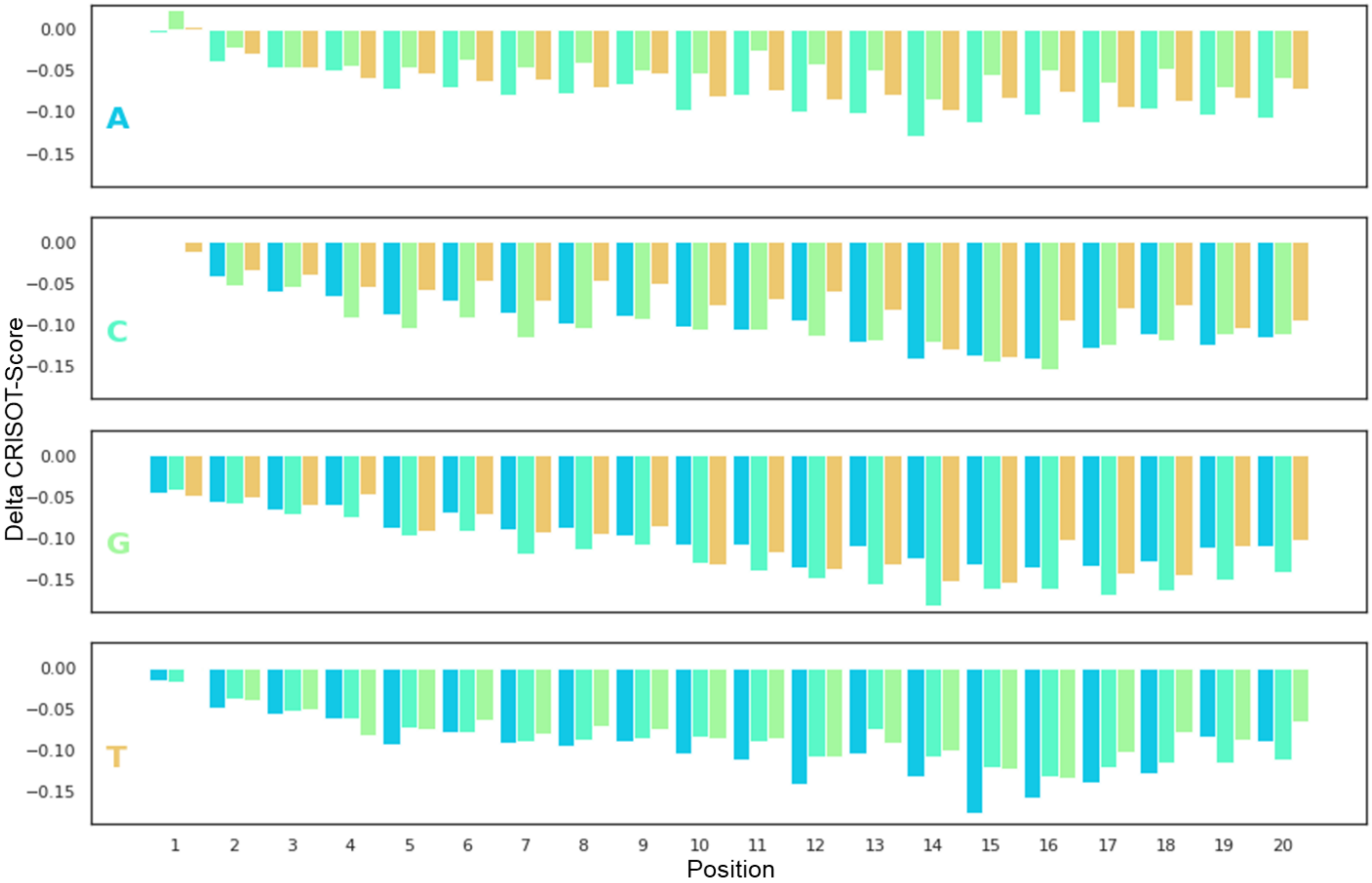


**Fig. S22 Summary of delta CRISOT-Score when the nucleotides of an sgRNA are mutated to different types.** The delta CRISOT-Score values are summarized from the CRISOT-Score map. The sequences are represented by the targeting sequences, i.e., the complementary sequences to the sgRNA. The A, C, G, and T nucleotides are colored blue, cyan, green and orange, respectively.

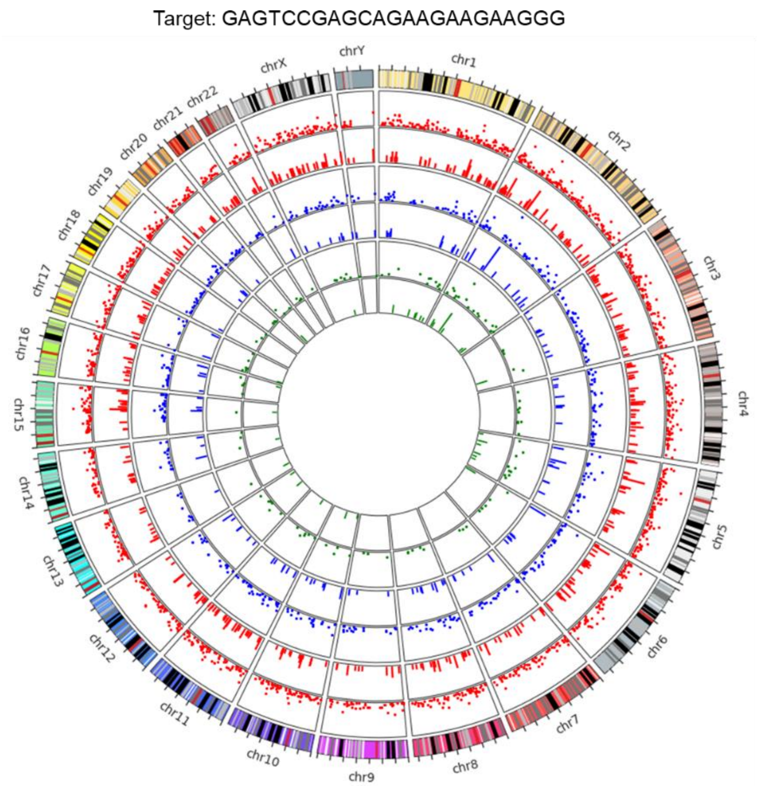


| | WT | A11>C | C6>A |
|---|---|---|---|
| CRISOT-Score | 0.947 | 0.867 | 0.876 |
| (0.65, 0.7] | 90 | 28 | 321 |
| (0.7, 0.75] | 22 | 4 | 82 |
| (0.75, 0.8] | 2 | 0 | 22 |
| (0.8, 1.0] | 1 | 0 | 5 |
| CRISOT-Spec | 0.364 | 0.720 | 0.140 |

**Fig. S23 Comparison of off-target profiles of the improperly mutated, origin and optimized sgRNAs.** The circos plots show the off-target sites with CRISOT-Score > 0.6. The scatter points indicate off-targets with CRISOT-Score values > 0.6, and the bars indicate those CRISOT-Score values > 0.65. Red, blue and green colors indicate the off-target sites of improperly mutated, origin and optimized sgRNAs, respectively. CRISOT-Score and CRISOT-Spec scores are in the right tables.

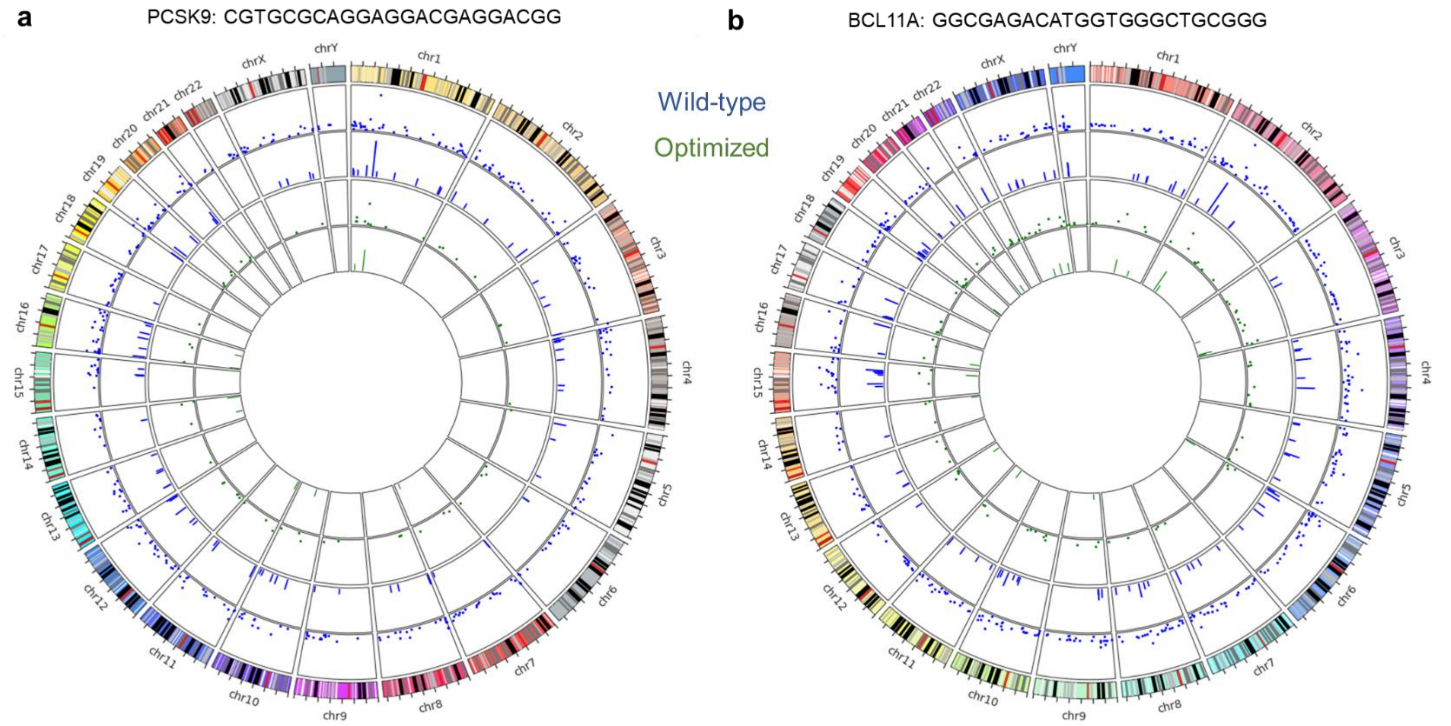


**Fig. S24 Comparison of CRISOT off-target profiles of the WT and optimized sgRNAs targeting the PCSK9 (a) and BCL11A (b) genes.** The points, lines and colors of the circos plots are the same as those in Fig. S23.

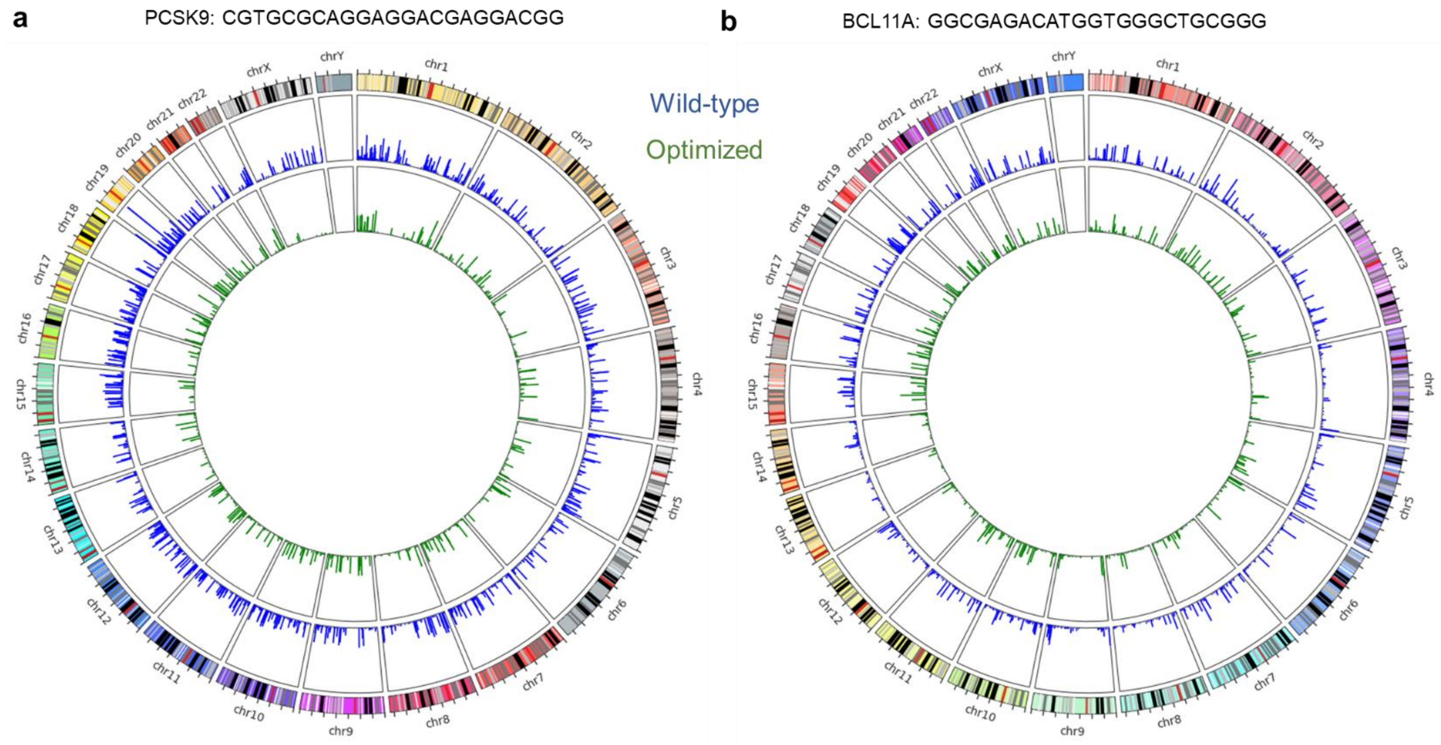


**Fig. S25 Comparison of WGS experimental off-target profiles of the WT and optimized sgRNAs targeting the PCSK9 (a) and BCL11A (b) genes.** Line length indicates the off-target frequencies. Blue and green colors indicate the off-target sites of WT and optimized sgRNAs, respectively.

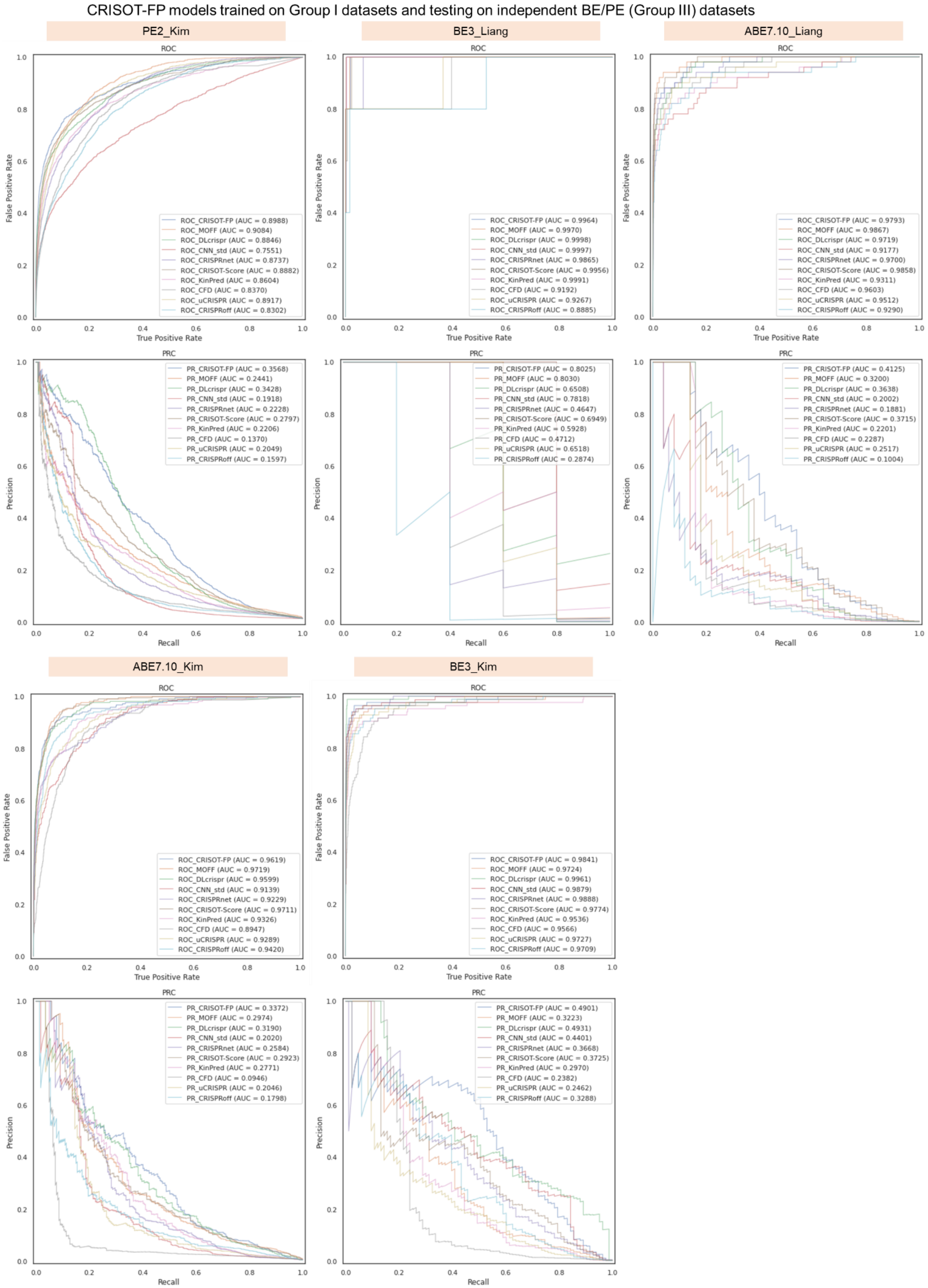


**Fig. S26 Comparison of CRISOT-FP and CRISOT-Score with existing off-target prediction methods in predicting off-target effects of BEs and PEs.** ROC and PR curves were shown. The names of BE and PE datasets were indicated in light-orange boxes. The CRISOT-FP models were trained on the Group I datasets.

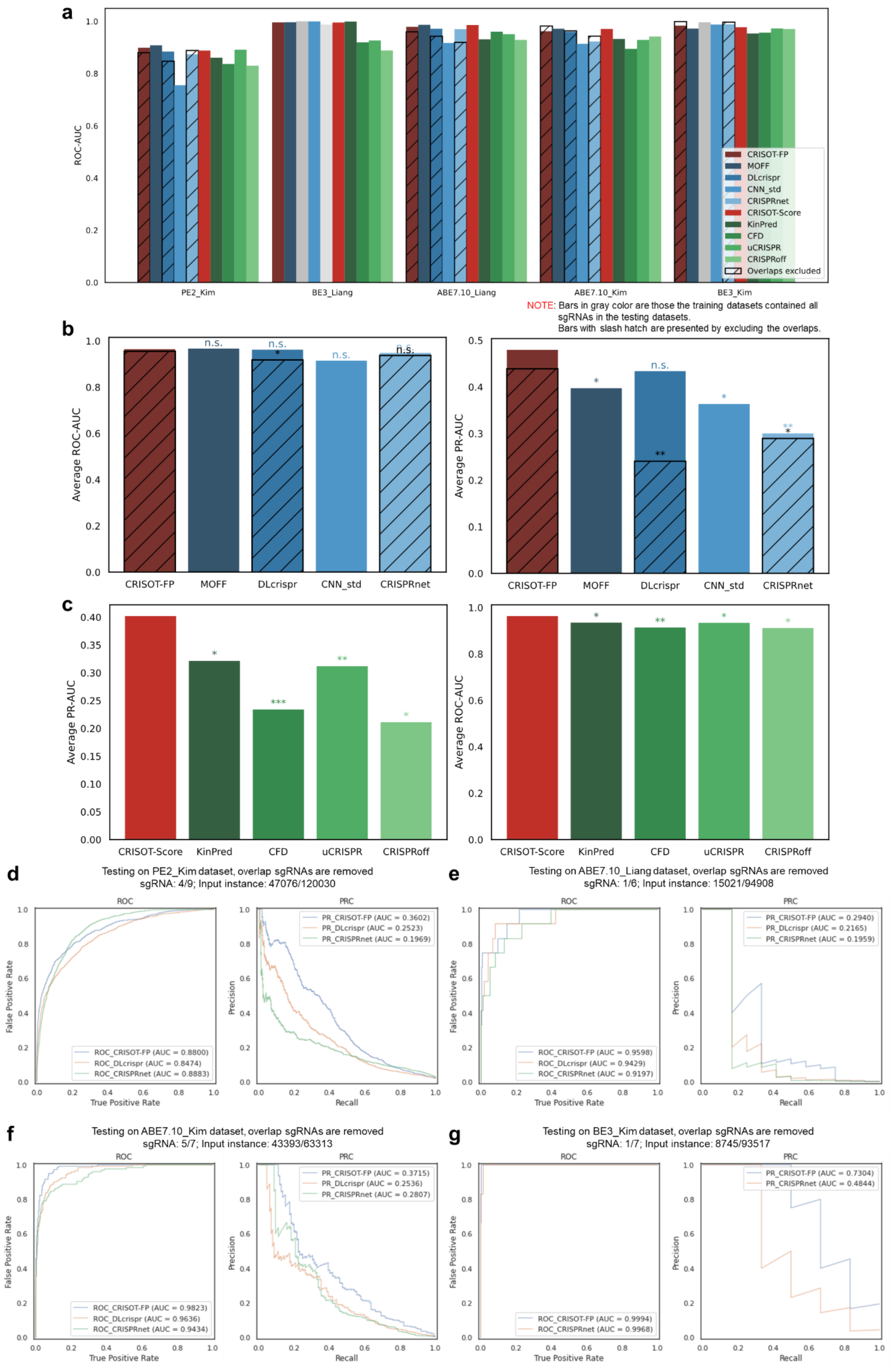


**Fig. S27 Comparison of CRISOT-FP and CRISOT-Score with the existing off-target prediction methods using the Group III datasets (base and prime editors). a**, The ROC-AUC results. The blue and green bars represent learning-based and hypothesis-driven methods, respectively. Bars in gray color are those the training datasets contained all sgRNAs in the

testing datasets. Bars with slash hatch are presented by excluding the overlapped sgRNAs. Of note, for fair comparisons, the same sgRNAs were removed from the testing datasets for CRISOT-FP. The overlapped sgRNAs are summarized in Supplementary Data 3. The CRISOT-FP models are trained on the Group I datasets, which are independent to the testing datasets. **b**, **c**, One-sided paired t-test of the PR-AUC and ROC-AUC, respectively. n.s.: not significant, *: p<0.05, **: p<0.01, ***: p<0.001, n=5. CRISOT-FP models were trained using the Group I datasets. The p-values for in **b** are 0.760, 0.366, 0.072, 0.052, 0.024, 0.087, 0.015 and 0.009, respectively. The p-values in **c** are 0.021, 0.007, 0.044, 0.019, 0.012, 0.0004, 0.002 and 0.022, respectively. The p-values exclluding overlaps are 0.022 and 0.122 for ROC-AUCs and 0.007 and 0.013 for PR-AUCs. **d** ~ **g**, ROC and PR curves for the datasets that partially overlap the training datasets of the comparing models. Overlap sgRNAs are removed from the testing datasets.

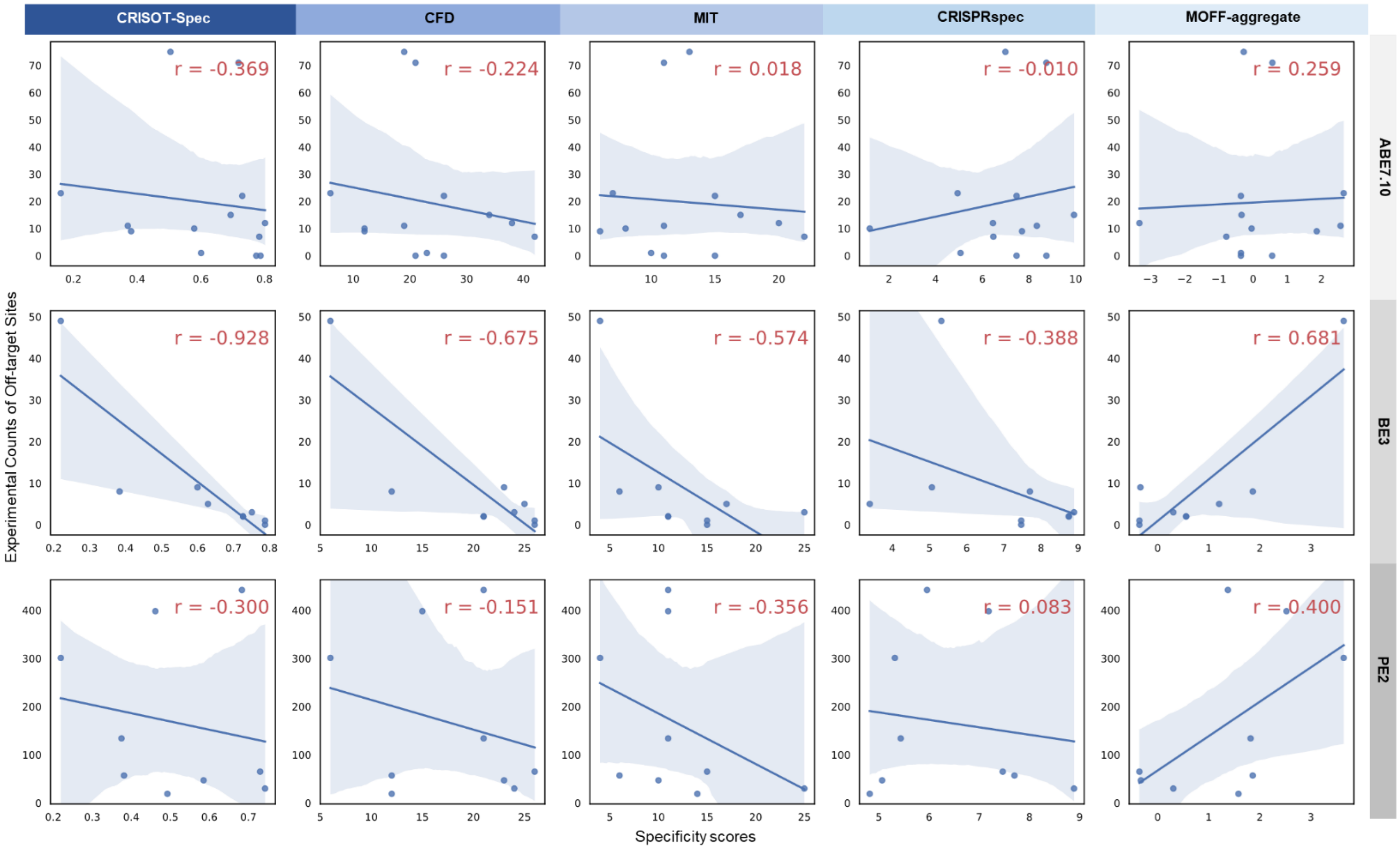


**Fig. S28 Correlations between specificity scores and the experimental counts of off-target sites of the Group III datasets (base and prime editors).** An sgRNA with a higher CRISOT-Spec score means higher specificity across the whole genome, thus stronger negative correlations between conventional specificity score and experimental off-target read fraction are expected. CRISOT-Spec is compared to three existing specificity scoring methods. The error bands represent the confidence intervals of 95% for the regression estimates.

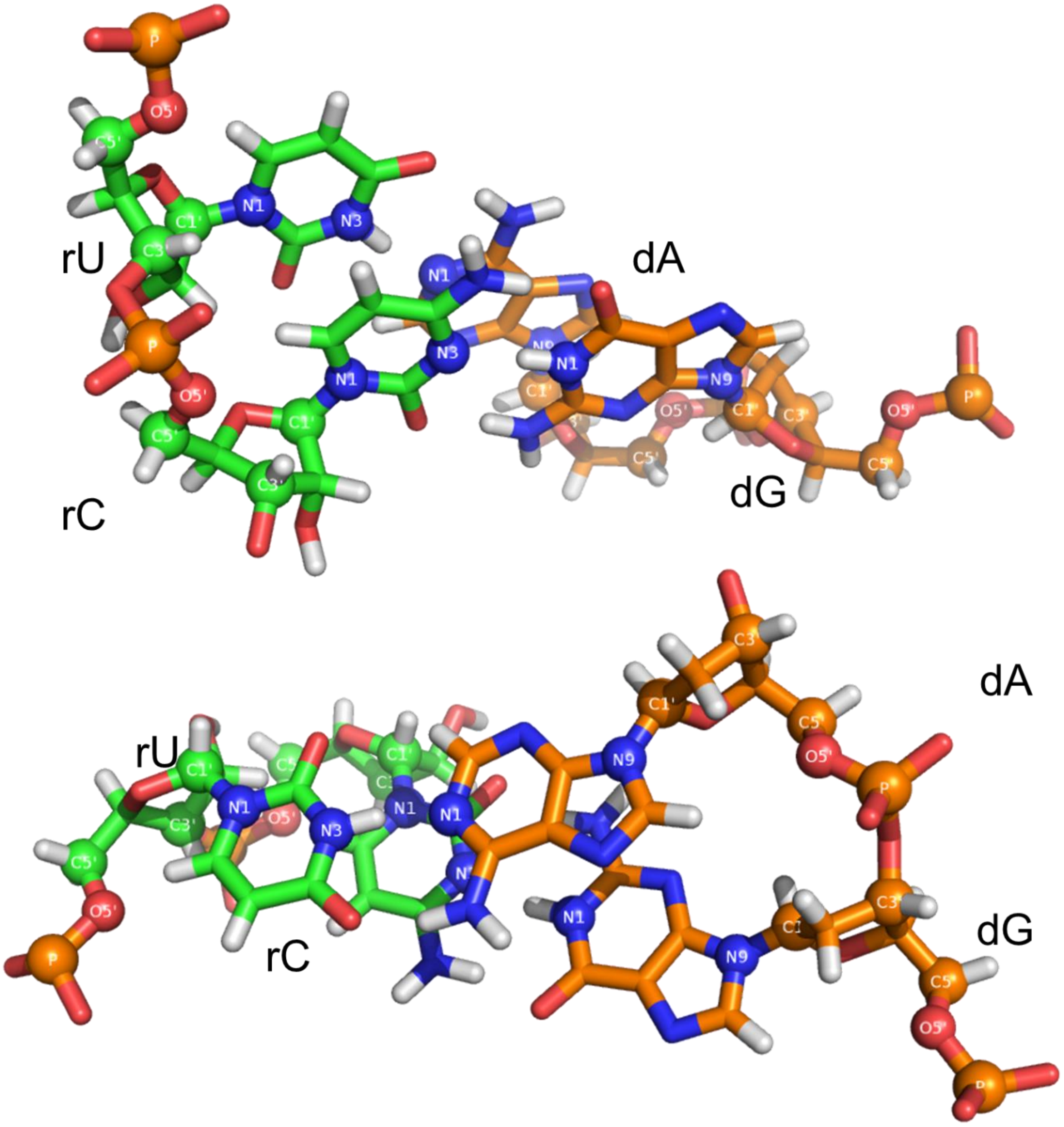


**Fig. S29 Selected atoms for calculations of distances, angles and dihedral angles.** Atom names of the selected atoms were indicated.

**Table S1. Sequences of dsODN-complementary oligonucleotides used in Guide-seq**

| Oligo name | Sequence 5′ to 3′ |
|---|---|
| oGS1 (dsODN FWD) | /5Phos/GTTTAATTGAGTTGTCATATGTTAATAACGGT*A*T |
| oGS2 (dsODN REV) | /5Phos/ATACCGTTATTAACATATGACAACTCAATTAA*A*C |

Note: /5Phos/ indicates 5′ phosphorylation. An asterisk indicates phosphorothioate linkage.

**Table S2. Nest primers used in the paper**

| Primer name | Forward | Reverse |
|---|---|---|
| BCL11A outside | CGGCTCTCCCACAATTCATCTT | AAAGGTGCGTGCTGTCTCA |
| BCL11A inside | TTCTCTGGAGTCTCCTTCTTTCT | TCTCTTTTACCTCGACTCTCGG |
| PCSK9 outside | CCAGGCAGTGAGACTGGCT | AAGATCGTGCCAAGCGAAGAGC |
| PCSK9 inside | CACGGCCTCTAGGTCTCCT | GAAACAGCACCGCACCG |
