# Supplementary material for: Genome-wide CRISPR off-target prediction and optimization using RNA-DNA interaction fingerprints
Source: Nat Commun. 2023 Nov 18;14:7521. doi: 10.1038/s41467-023-42695-4 (PMC10657421; doi:10.1038/s41467-023-42695-4)
Supplement: Supplementary file 3 — Description of Additional Supplementary Files [file 41467_2023_42695_MOESM3_ESM.pdf]

Title: Supplementary Data 1
Description: CRISOT-FP features and encoding examples.

Title: Supplementary Data 2
Description: SHAP values and feature importance values.

Title: Supplementary Data 3
Description: Information for the overlapped sgRNAs.

Title: Supplementary Data 4
Description: CRISOT-Opti optimization results.

Title: Supplementary Data 5
Description: Genome-wide off-target results of the Guide-seq experiments.

Title: Supplementary Data 6
Description: Genome-wide off-target results of the WGS experiments.

Title: Supplementary Data 7
Description: Model fine-tuning and configuration.
