# Supplementary material for: Genome-wide CRISPR off-target prediction and optimization using RNA-DNA interaction fingerprints
Source: Nat Commun. 2023 Nov 18;14:7521. doi: 10.1038/s41467-023-42695-4 (PMC10657421; doi:10.1038/s41467-023-42695-4)
Supplement: Supplementary file 11 — Reporting Summary [file 41467_2023_42695_MOESM11_ESM.pdf]

nature portfolio

Corresponding author(s): Qi Liu

Last updated by author(s): Oct 19, 2023

Reporting Summary

Nature Portfolio wishes to improve the reproducibility of the work that we publish. This form provides structure for consistency and transparency in reporting. For further information on Nature Portfolio policies, see our Editorial Policies and the Editorial Policy Checklist.

Statistics

For all statistical analyses, confirm that the following items are present in the figure legend, table legend, main text, or Methods section.

n/a	Confirmed	
☐	☒	The exact sample size (n) for each experimental group/condition, given as a discrete number and unit of measurement
☐	☒	A statement on whether measurements were taken from distinct samples or whether the same sample was measured repeatedly
☐	☒	The statistical test(s) used AND whether they are one- or two-sided<br>*Only common tests should be described solely by name; describe more complex techniques in the Methods section.*
☒	☐	A description of all covariates tested
☒	☐	A description of any assumptions or corrections, such as tests of normality and adjustment for multiple comparisons
☐	☒	A full description of the statistical parameters including central tendency (e.g. means) or other basic estimates (e.g. regression coefficient) AND variation (e.g. standard deviation) or associated estimates of uncertainty (e.g. confidence intervals)
☐	☒	For null hypothesis testing, the test statistic (e.g. F, t, r) with confidence intervals, effect sizes, degrees of freedom and P value noted<br>*Give P values as exact values whenever suitable.*
☒	☐	For Bayesian analysis, information on the choice of priors and Markov chain Monte Carlo settings
☒	☐	For hierarchical and complex designs, identification of the appropriate level for tests and full reporting of outcomes
☐	☒	Estimates of effect sizes (e.g. Cohen's d, Pearson's r), indicating how they were calculated

Our web collection on statistics for biologists contains articles on many of the points above.

Software and code

Policy information about availability of computer code

Data collection	gmx_MMPBSA v1.2.0<br>dnaMD 2018.12<br>pytraj 2.0.2<br>MDAnalysis 2.0.0<br>MDTraj 1.9.3<br>Cas-OFFinder 2.4
Data analysis	XGBoost 1.7.3<br>SHAP 0.39.0<br>seaborn 0.11.2<br>sklearn 0.24.1<br>Guide-seq 1.0

For manuscripts utilizing custom algorithms or software that are central to the research but not yet described in published literature, software must be made available to editors and reviewers. We strongly encourage code deposition in a community repository (e.g. GitHub). See the Nature Portfolio guidelines for submitting code & software for further information.

Data

Policy information about availability of data

All manuscripts must include a data availability statement. This statement should provide the following information, where applicable:

- Accession codes, unique identifiers, or web links for publicly available datasets
- A description of any restrictions on data availability
- For clinical datasets or third party data, please ensure that the statement adheres to our policy

Source data are provided with this paper. All data supporting the findings of this study are available in the paper and the Supplementary Information files. Additional original data that support the findings are available from the corresponding author upon request. The Guide-seq sequencing data for sgRNAs targeting EMX1 generated in this study have been deposited in the NCBI Sequence Read Archive under accession number PRJNA785744 [https://www.ncbi.nlm.nih.gov/bioproject/?term=PRJNA785744], and the WGS data for sgRNAs targeting PCSK9 and BCL11A generated in this study have been deposited under accession number PRJNA921906 [https://www.ncbi.nlm.nih.gov/bioproject/?term= PRJNA921906]. Supplementary data for the features, feature importance values, overlapped sgRNAs, sgRNA optimization, off-target data, et al. are available as Supplementary Data 1-7. The training/testing datasets, have been deposited in the Zenodo repository [https://doi.org/10.5281/zenodo.8420032].

Human research participants

Policy information about studies involving human research participants and Sex and Gender in Research.

Reporting on sex and gender	N/A
Population characteristics	N/A
Recruitment	N/A
Ethics oversight	N/A

Note that full information on the approval of the study protocol must also be provided in the manuscript.

Field-specific reporting

Please select the one below that is the best fit for your research. If you are not sure, read the appropriate sections before making your selection.

☒ Life sciences ☐ Behavioural & social sciences ☐ Ecological, evolutionary & environmental sciences

For a reference copy of the document with all sections, see nature.com/documents/nr-reporting-summary-flat.pdf

Life sciences study design

All studies must disclose on these points even when the disclosure is negative.

Sample size	Sample sizes were determined based on literature precedence for genome editing experiments.
Data exclusions	No data were excluded.
Replication	Three independent MD runs were performed for each structural complex; One standard Guideseq assay was performed for each sgRNA; Three independent WGS assays were performed for each sgRNA.<br>All attempts for replications were successful.
Randomization	Due to the small sample, randomization was not relevant for this study. Covariates were controlled for by running controls in parallel whenever applicable.
Blinding	Blinding was not relevant to our study because in general, based on the prior experience of other groups in the field, these types of assays do not require blinding.

Reporting for specific materials, systems and methods

We require information from authors about some types of materials, experimental systems and methods used in many studies. Here, indicate whether each material, system or method listed is relevant to your study. If you are not sure if a list item applies to your research, read the appropriate section before selecting a response.

Materials & experimental systems

n/a	Involved in the study
☒	☐ Antibodies
☐	☒ Eukaryotic cell lines
☒	☐ Palaeontology and archaeology
☒	☐ Animals and other organisms
☒	☐ Clinical data
☒	☐ Dual use research of concern

Methods

n/a	Involved in the study
☒	☐ ChIP-seq
☒	☐ Flow cytometry
☒	☐ MRI-based neuroimaging

Eukaryotic cell lines

Policy information about cell lines and Sex and Gender in Research

Cell line source(s)	HEK293 and HEK293T cells were obtained from Cell bank of Shanghai Institute of Biochemistry and Cell biology, Chinese Academy of Sciences.
Authentication	Cell lines were authenticated with STR profiling by supplier.
Mycoplasma contamination	All cell lines were tested negative for mycoplasma contamination.
Commonly misidentified lines (See ICLAC register)	No commonly misidentified cell lines were used in this study.
